# Effect and possible mechanisms of saponins in Chinese herbal medicine exerts for the treatment of myocardial ischemia-reperfusion injury in experimental animal: a systematic review and meta-analysis

**DOI:** 10.3389/fcvm.2023.1147740

**Published:** 2023-07-26

**Authors:** Jiahao Sun, Jiarong Fan, Fan Yang, Xin Su, Xinye Li, Li Tian, Can Liu, Yanwei Xing

**Affiliations:** ^1^ Yanqing Hospital of Beijing Chinese Medicine Hospital, Beijing, China; ^2^ Clinical Department of Integrative Traditional Chinese and Western Medicine, Beijing University of Chinese Medicine, Beijing, China; ^3^ Guang'anmen Hospital, China Academy of Chinese Medical Sciences, Beijing, China; ^4^ Sun Yat-sen University Cancer Center, Guangzhou, China

**Keywords:** meta-analysis, saponins, traditional Chinese medicine, myocardial ischemia-reperfusion injury, experimental animal model, infarct size, cardiac function

## Abstract

**Introduction:**

Preventing ischemia-reperfusion injury is the main direction of myocardial infarction treatment in the convalescent stage. Some studies have suggested that saponins in Traditional Chinese medicine (TCM) preparations can protect the myocardium by various mechanisms. Our meta-analysis aims to evaluate the efficacy of TCM saponins in treating myocardial ischemia-reperfusion injury (MIRI) and to summarize the potential molecular mechanisms further.

**Methods:**

We conducted a literature search in six electronic databases [Web of Science, PubMed, Embase, Cochrane Library, Sinomed, China National Knowledge Infrastructure (CNKI)] until October 2022.

**Results:**

Seventeen eligible studies included 386 animals (254 received saponins and 132 received vehicles). The random effect model is used to calculate the combined effect. The effect size is expressed as the weighted average difference (WMD) and 95% confidence interval (CI). Compared with placebo, saponins preconditioning reduced infarct size after MIRI significantly (WMD: −3.60,95% CI: −4.45 to −2.74, *P* < 0.01, *I*^2^: 84.7%, *P* < 0.001), and significantly increased EF (WMD: 3.119, 95% CI: 2.165 to 4.082, *P* < 0.01, *I*^2^: 82.9%, *P* < 0.0 L) and FS (WMD: 3.157, 95% CI: 2.218 to 4.097, *P* < 0.001, *I*^2^: 81.3%, *P* < 0.001).

**Discussion:**

The results show that the pre-administration of saponins from TCM has a significant protective effect on MIRI in preclinical studies, which provides an application prospect for developing anti-MIRI drugs with high efficiency and low toxicity.

## 1. Introduction

Timely percutaneous coronary intervention (PCI) remains by far the most effective treatment for the salvage of acute myocardial infarction (1). However, subsequent ischemia/reperfusion (I/R) injury can still lead to massive loss of cardiomyocytes, thereby exacerbating cardiac dysfunction (2). Now, MIRI has become a vital issue in clinical practice, attracting extensive attention from researchers. MIRI involves multiple mechanisms, such as inflammation, oxidative stress, mitochondrial damage, and calcium overload (3). Recently, researchers have begun to focus on ferroptosis, apoptosis, and autophagy during MIRI. Several drug and non-drug therapies have emerged to treat I/R injury (4). However, given that treatments targeting I/R injury would be used only once in most patients, their development, compared to drugs that require longer-term use, is at an obvious disadvantage.

TCM emphasizes the holistic concept. The herbal prescriptions are usually complex, targeting multiple pathways (5, 6). In TCM, the causes of MIRI include Qi deficiency, blood stasis, and phlegm resistance (7–9). According to TCM theories, doctors mainly use herbs that nourish qi, promote blood circulation, remove blood stasis, and expel phlegm. Recently, multiple studies have found that saponins from traditional Chinese herbs have sound anti-MIRI effects (10–12). The mechanisms include regulating calcium homeostasis and energy metabolism and inhibiting inflammation and oxidative stress (13–16). Saponins mainly contain steroid saponins and triterpenoid saponins. Due to their structural specificity, saponins have different MIRI protection mechanisms (17). Therefore, we conducted a comprehensive and systematic meta-analysis of all relevant articles to evaluate the effectiveness and summarize the possible means of TCM saponin extract intervention in alleviating I/R injury.

## 2. Methods

### 2.1. Data sources and search strategies

We searched Web of Science, PubMed, Embase, Cochrane Library, Sinomed, and China National Knowledge Infrastructure (CNKI) for articles on the cardioprotective effects of saponins in myocardial ischemia-reperfusion injury. The search terms were “myocardial ischemia-reperfusion injury,” “myocardial I/R injury,” “myocardial ischemia-reperfusion injury,” “myocardial ischemia-reperfusion injury,” “saponins,” “ginsenosides,” “Holothurin,” and “Quillaja Saponins,”. The publication time was limited to before October 2022. Two reviewers (Fan Yang and Xin Su) independently assessed articles for eligibility, and disagreements were resolved by discussion with the corresponding author.

### 2.2. Inclusion and exclusion criteria

Inclusion criteria: (a) Type of study: In vivo animal studies. (b) Interventions and controls: Saponin extracts derived from traditional Chinese herbs compared with placebo treatment. (c) Outcomes: At least one of the following outcome measures should be reported: Infarct size [infarct size expressed as a percentage of infarct area over the area at risk (AAR)], left ventricle ejection fraction percentage (LVEF), left ventricle fractional shortening percentage (LVFS).

Exclusion criteria: (a) Duplicate reports. (b) Studies with incomplete data and unclear outcome indicators. (c) Studies where the outcome indicators could not be transformed into mergeable data. (d) Studies using incorrect statistical methods that could not be corrected.

### 2.3. Data extraction

Two researchers (Jiahao Sun and Jiarong Fan) independently extracted baseline information (i.e., sample size, country, author, and year), animal characteristics (e.g., age, weight, strain, or species), detailed treatments of included studies Strategy (such as the type of saponin, source plant, dose, method and time of administration) and methods for measuring myocardial infarction size after myocardial I/R injury.

### 2.4. Quality assessment

The quality of included studies was assessed and scored by two researchers (Yang Fan and Su Xin) according to the CAMARADES(Collaborative Approach to Meta Analysis and Review of Animal Data from Experimental Stroke) list (18). For each article, we scored 1 point for each of the following terms: statement of a potential conflict of interest, sample size calculation, random allocation to groups, a peer-reviewed publication, compliance with animal welfare regulations, and blinded outcomes assessment. We define 1–2 points as high risk, 3–4 points as medium risk, and 5–6 points as low risk. Disagreements were resolved through discussions with the corresponding author.

### 2.5. Data statistical and analysis

This meta-analysis used continuous variables as mean and standard deviation. We calculated pooled effect sizes using random effects models. Effect sizes were expressed as weighted mean differences (WMD) with 95% confidence intervals (CI). To assess heterogeneity, we used the Q statistic and the *I*^2^ statistic for quantifying. Publication bias was evaluated initially by funnel plots and further detected by Egger's test. If there were significant heterogeneity among studies (*p* < 0.10), the analysis would be performed by deleting each study, *post hoc* subgroup analysis (i.e., duration of reperfusion, study type, species, and schedule of preconditioning), and trim-and-fill method to achieve sensitivity analysis. Univariate regression (i.e., study type, species, states, sample size, route of administration and duration of reperfusion, and temporal regimen of preconditioning) was proposed to explore potential heterogeneity sources. For all results except heterogeneity, *P *< 0.05 was considered statistically significant. We used STATA version 15.1 (STATA Corporation, CollegeStation, TX, USA) for statistical analyses and graphs.

## 3. Results

### 3.1. Characteristics of included studies

**Figure 1:**
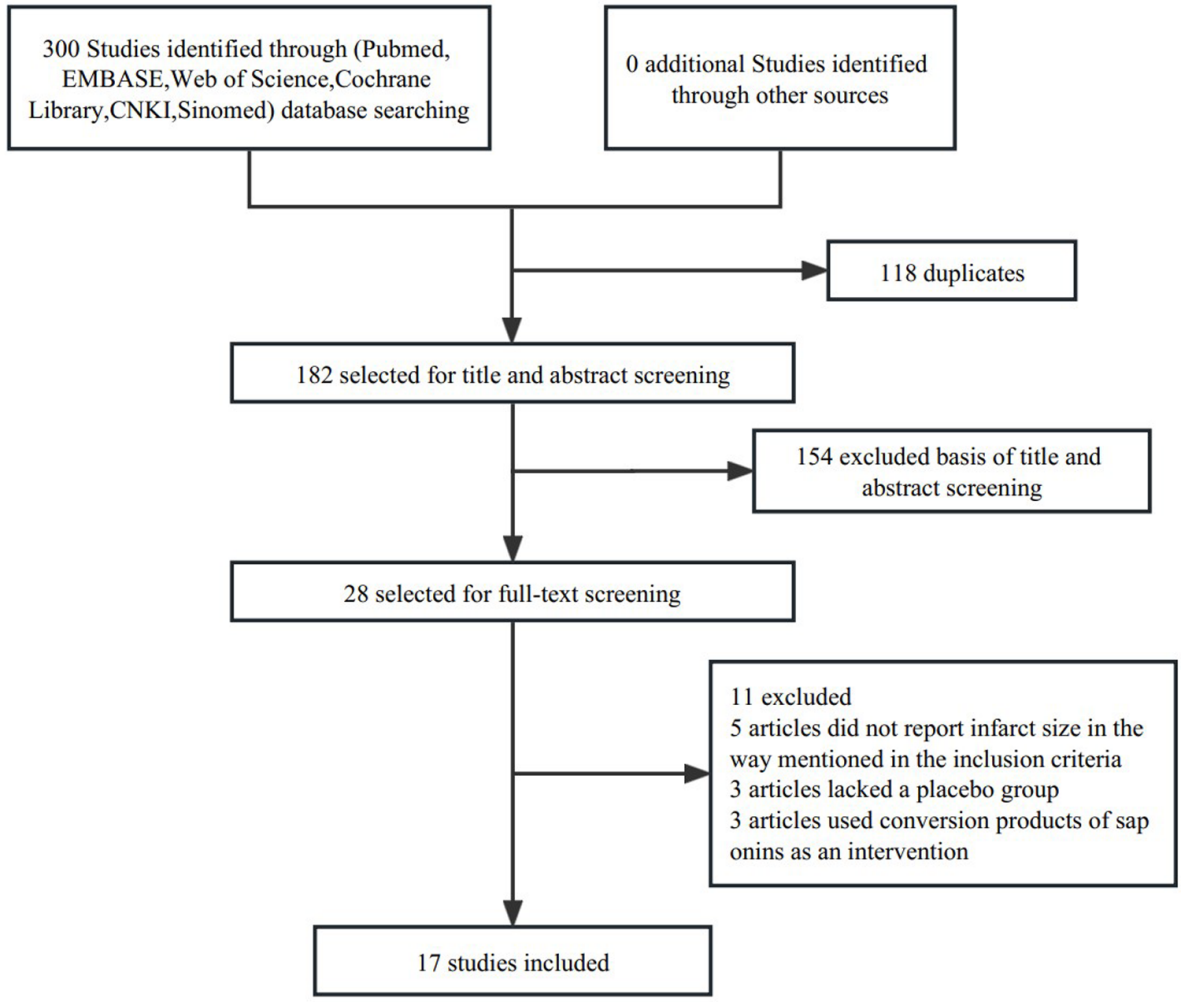
Flow diagram of the study inclusion.

We obtained 300 related articles (PubMed: 64, EMBASE: 47, Web of Science: 55, Cochrane Library: 1, CNKI: 91, Sinomed: 42) by searching in databases, and 182 articles were retained after removing duplicate articles, among which 154 were excluded after the title and abstract screening, and 28 were eligible for full-text review. Five articles did not report infarct size as mentioned in the inclusion criteria, three lacked a placebo group, and three used saponin conversion products as an intervention. Finally, 17 studies of 386 animals (254 in the saponin treatment group and 132 in the control group) met the predetermined inclusion criteria and were eventually included in our meta-analysis (Figure 1) (4, 5, 10, 13, 17, 19–30). Table 1 shows comprehensive information for each study. All studies used mice (C57BL/6) and rats (Sprague-Dawley or Wistar) to investigate the cardioprotective effects of saponins. All studies measured infarct size by Evans blue/TTC double staining. Saponins were administered orally or intravenously, or intraperitoneally. In most studies, the administration began a few days before myocardial ischemia, and some studies were administered within half an hour before ischemia or during reperfusion. All 17 studies were conducted in China and were published between 2013 and 2022.

**Table 1:** Baseline characteristics of studies.

Author	Year	State	Compound	Major plant source	Treatment	Sex	Species	Weight	Sample Size		I/R Duration
									Control	Saponin	
He et al.	2018	China	Saponins from Rhizoma Panacis Majoris	*Rhizoma Panacis Majoris*	200 mg/kg, p.o., 14 days/30 min before I/R	Male	Rats, Wistar	200 g ± 20 g	8	8	30 min/2 h
Huang et al.	2021	China	Saponin astragaloside IV	*Astragalus membranaceus*	20 mg/kg; i.p., during reperfusion	Male	Mice, C57BL/6	20–25 g	6	6	30 min/4 h
Wang et al.	2021	China	Calenduloside E	*Aralia elata (Miq.)*	7.5/15/30 mg/kg,p.o., 3 days before I/R	Male	Rats, SD	270–280 g	5	5	30 min/48 h
Wang et al.	2018	China	Total Saponins of Aralia Elata (Miq) Seem	*Aralia elata (Miq.)*	25/50/100 mg/kg, p.o., 5 days before I/R	Male	Rats, SD	260–300 g	6	6	30 min/48 h
Wei et al.	2019	China	Astragaloside IV	*Astragalus membranaceus*	2.5/5 mg/kg, p.o., 7 days before I/R	Male	Rats, SD	220–240 g	11	10	30 min/2 h
					10 mg/kg, p.o., 7 days before I/R	Male	Rats, SD	220–240 g	11	11	30 min/2 h
Wu et al.	2022	China	Ilexsaponin Ⅰ	*Ilex pubescens Hook*	30 mg/kg, i.p., 15 min before reperfusion	Male	Rats, SD	250 ± 10 g	5	5	30 min/24 h
Yan et al.	2015	China	Total saponins extracted from Aralia taibaiensis	*Aralia taibaiensis*	60/120/240 mg/kg.p.o., 7 days before I/R	Male	Rats, SD	250 ± 30 g	8	8	30 min/3 h
Zhang et al.	2017	China	Ilexsaponin A	*Ilex pubescens Hook. et Arn.*	10/40 mg/kg, i.p.,10 min before I/R	Male	Rats, SD	280–320 g	8	8	30 min/4 h
Zhu et al.	2017	China	Apigenin-7-O-*β*-D-(-6″-p-coumaroyl)-glucopyranoside	*Clematis tangutica*	1/5/10 mg/kg, i.p., 3 days before I/R	Male	Mice, C57BL/6	20–22 g	6	6	30 min/24 h
Wang et al.	2020	China	Calenduloside E	*Aralia elata (Miq.)*	15 mg/kg,p.o., 3 days before I/R	Male	Rats, SD	230–250 g	6	6	30 min/48 h
Su et al.	2022	China	Gypenoside XVII	*Gynostemma pentaphyllum*	30/60 mg/kg,p.o., 14 days before I/R	Male	Mice, C57BL/6J	20–22 g	10	10	120 min/unknown
Lv et al.	2021	China	Tubeimoside Ⅰ	*Bulbus Bolbostemmae Paniculati*	4 mg/kg, i.p.,15 min after I/R	Male	Mice, C57BL/6	Unknown	6	6	30 min/24 h
Yu et al.	2021	China	Panax quinquefolius L. saponins	*American Panax ginseng*	70/100 mg/kg. p.o., 7 days before I/R	Male/Female	Rats, Wistar	210–230 g	6	6	120 min/2 h
Yao et al.	2019	China	Total saponins from Clematis tangutica	*Clematis tangutica*	50/100/200 mg/kg,i.p., 7 days before I/R	Male	Rats, SD	250 ± 30	10	10	30 min/3 h
Sun et al.	2014	China	Panax quinquefolius saponins	*Panax quiquefolium L.*	100/300 mg/kg,i.g., 15 days before I/R	Male/Female	Rats, SD	200 ± 20	8	8	30 min/3 h
Wang et al.	2013	China	Panax quinquefolium saponins	*Panax quiquefolium L.*	270 mg/kg,i.g.,42 days before I/R	Male/Female	Rats, SD	150 ± 20	3	3	45 min/24 h
Li et al.	2014	China	Senegenin	*Polygala*	30 mg/kg,p.o., 10 min before I/R	Male	Mice, C57BL/6	3 months	20	20	45 min/3 h

SD, Sprague-Dawley; M, male; i.p., intraperitoneal injection; i.v., intravenous injection; p.o., orally treated; I/R, ischemia/reperfusion injury; TTC, triphenyl tetrazolium chloride.

### 3.2. Quality assessment of studies

The quality of the included studies was evaluated in Table 2. Most studies (82.4%) were scored 3 to 4, indicating that the data were generally reliable and the risk of bias was acceptable. Three studies were judged as having a high risk of bias.

**Table 2:** The quality of included studies.

Studies	Year	A	B	C	D	E	F	Score
Zhu et al.	2017	Y	N	N	N	Y	Y	3
Huang et al.	2021	Y	N	N	N	Y	Y	3
Wei et al.	2019	Y	Y	N	N	Y	N	3
Wang et al.	2020	Y	Y	N	N	Y	Y	4
Wang et al.	2021	Y	Y	N	N	Y	Y	4
Su et al.	2022	Y	N	N	N	Y	Y	3
Wu et al.	2022	Y	Y	N	N	Y	Y	4
Zhang et al.	2017	Y	Y	N	N	Y	N	3
Yu et al.	2020	Y	Y	N	N	Y	Y	4
He et al.	2018	Y	Y	N	N	Y	Y	4
Yan et al.	2015	Y	Y	N	N	Y	N	3
Wang et al.	2018	Y	N	N	N	Y	Y	3
Lv et al.	2021	Y	Y	N	N	Y	Y	4
Li et al.	2014	Y	Y	N	N	N	N	2
Wang et al.	2013	Y	Y	N	N	Y	N	3
Sun et al.	2014	Y	Y	N	N	N	N	2
Yao et al.	2019	Y	Y	N	N	N	N	2

A, peer-reviewed publication; B, random allocation to groups; C, blinded outcomes assessment; D, sample size calculation; E, compliance with animal welfare regulations; F, a statement of a potential conflict of interest; Y, yes; N, no.

### 3.3. Infarct size

**Figure 2:**
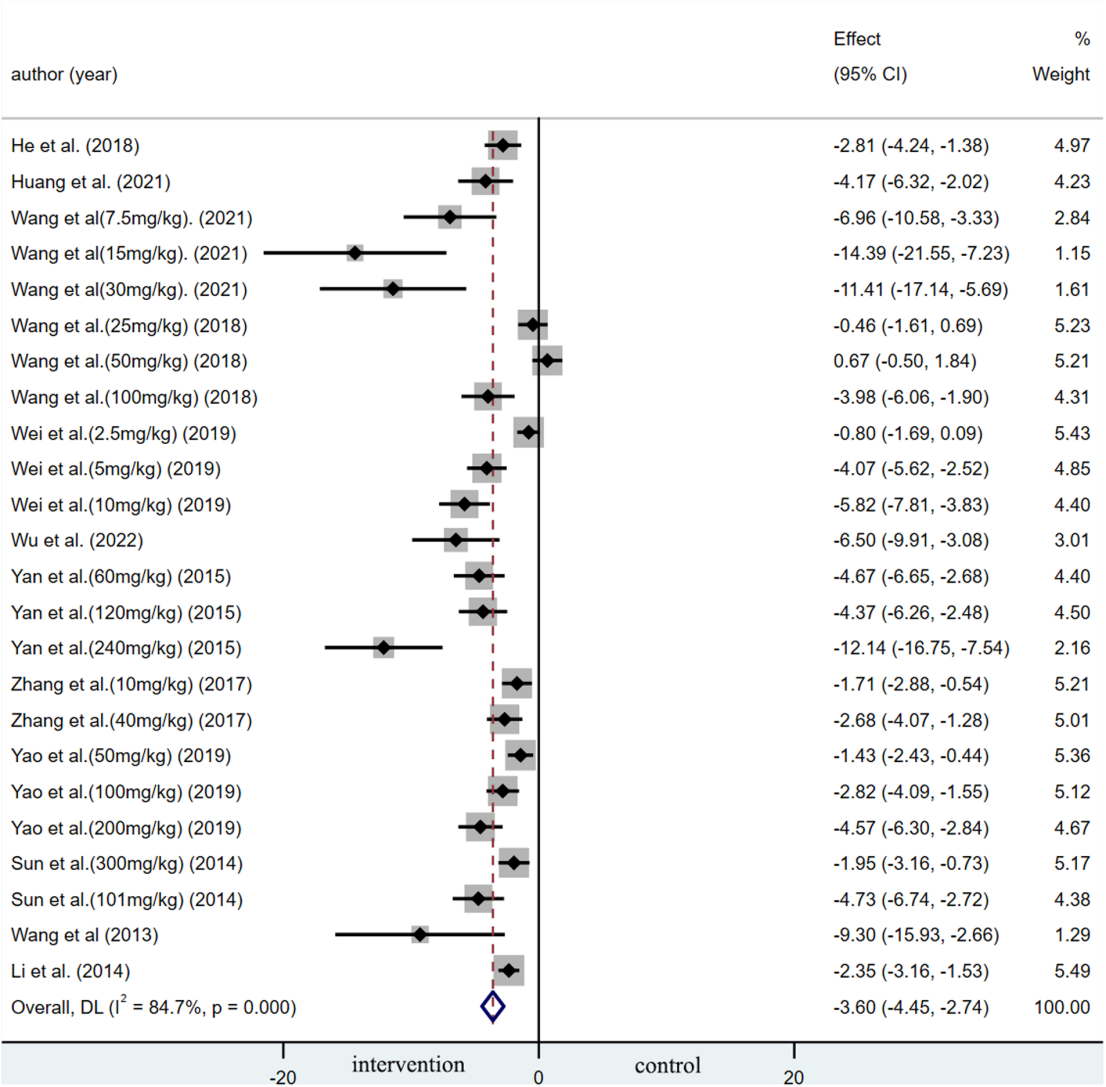
Summary WMD of infarct size for saponins treatment vs. vehicle in myocardial I/R injury.

**Figure 3:**
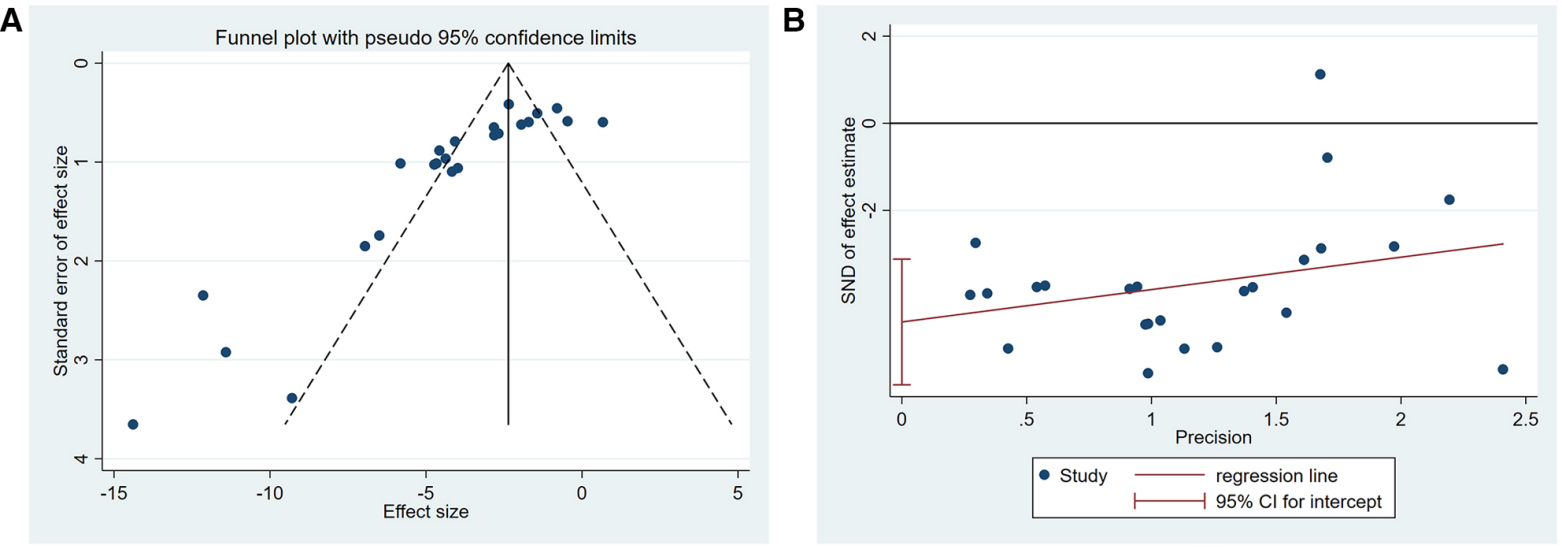
Publication bias among the studies reporting the infarct size. (**A**) Funnel plot, (**B**) Egger's test.

**Figure 4:**
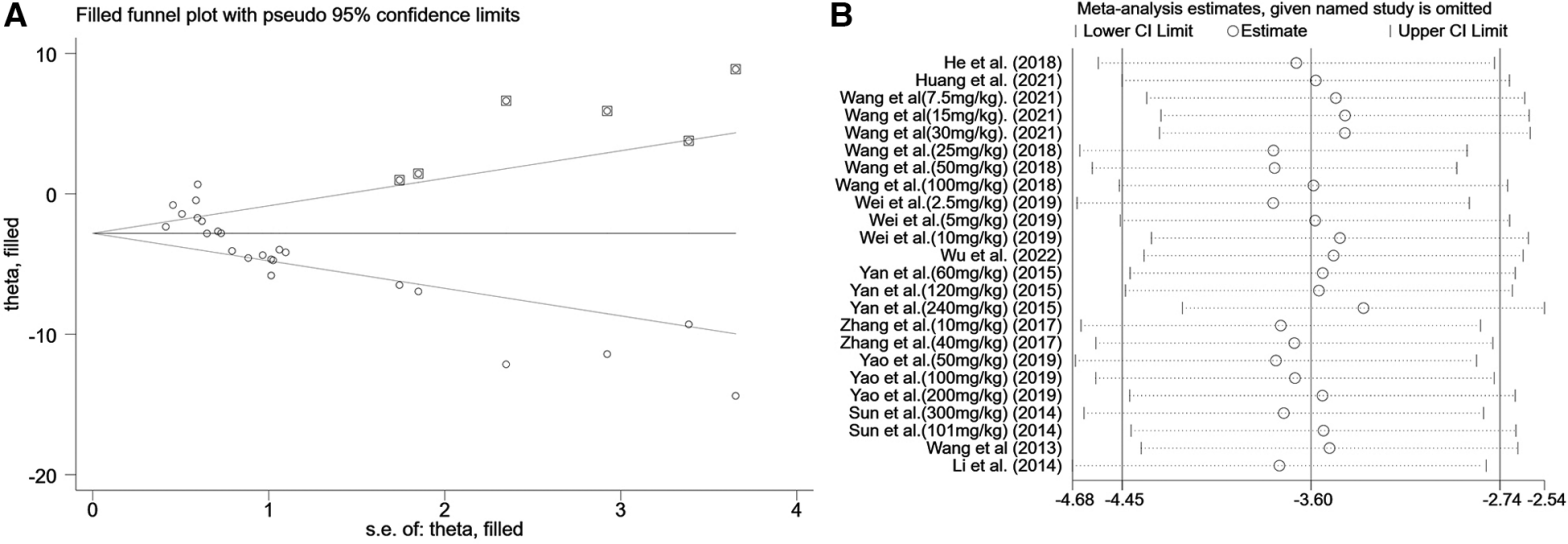
Sensitivity analysis of summary WMD of infarct size for saponins treatment vs. vehicle in myocardial I/R injury. (**A**) Filled funnel plot with pseudo 95% confidence limits. (**B**) Pooled effect sizes and 95% confidence limits after each study were omitted.

**Figure 5:**
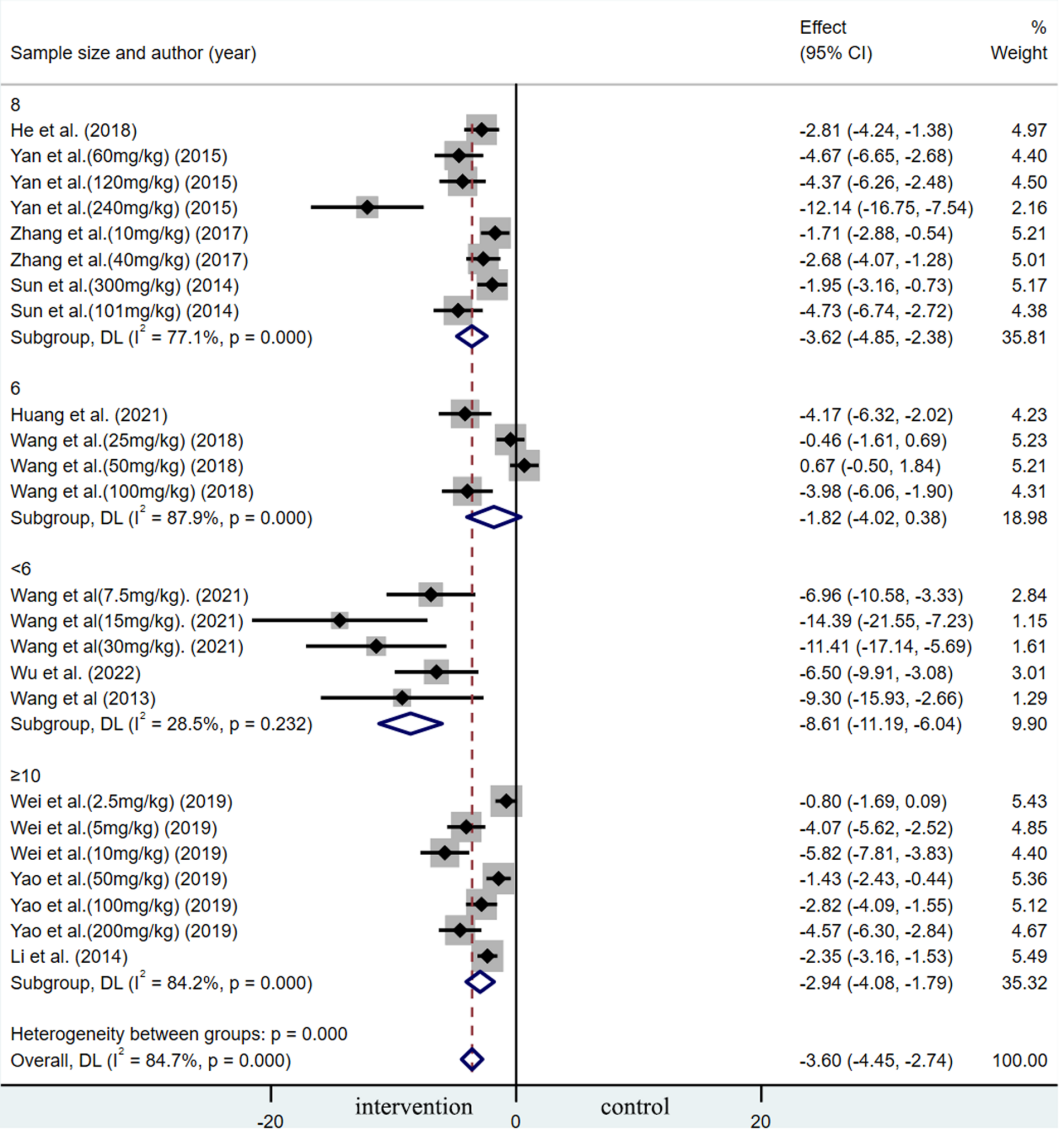
Subgroup analysis of summary WMD of infarct size for saponins treatment vs. vehicle in MIRI.

A summary analysis of 24 arms with 290 animals (intervention group = 192, control group = 98) showed the consistent effect of saponins on myocardial infarction size (Figure 2). Random effect model analysis showed that compared with placebo treatment, saponins significantly reduced infarct size (WMD: −3.60, 95% CI: −4.45 to−2.74, *P *< 0.01). There is a large amount of heterogeneity between studies (*I*^2^: 84.7%, *P *< 0.001). The funnel plot and Egger test (*P *< 0.001) showed the existence of publication bias (Figure 3). After introducing possibly unpublished studies through the trim and fill method, the results were steady (WMD: −2.81, 95% Cl: −3.69 to−1.93, *P *< 0.001), and the systematic deletion of each study did not affect the summary WMD and corresponding *P* values (Figure 4), which means that the beneficial effect of saponins in myocardial protection is stable. Subgroup analysis to explore the sources of heterogeneity between studies showed that sample size was a possible source of heterogeneity (Figure 5). Univariate regression failed to reveal any significant correlation between research covariables and effect sizes (Table 3).

**Table 3:** Meta-regression of pooled estimates of infarct size.

Covariates	exp(b)	95% CI	*P* value
Year	0.8567716	0.4870931 to 1.507017	0.576
Sample size	1.277729	0.8545751 to 1.910412	0.220
Species	0.5089549	0.033855 to 7.651319	0.610
Way of administration	2.802122	0.2908092 to 27.00014	0.356
Dosage	0.9908395	0.9756434 to 1.006272	0.230
Time of administration	0.9701195	0.7321618 to 1.285415	0.825
Time of reperfusion	0.9799072	0.9108849 to 1.05416	0.570

### 3.4. Cardiac function

#### 3.4.1. Left ventricular ejection fraction percentage

**Figure 6:**
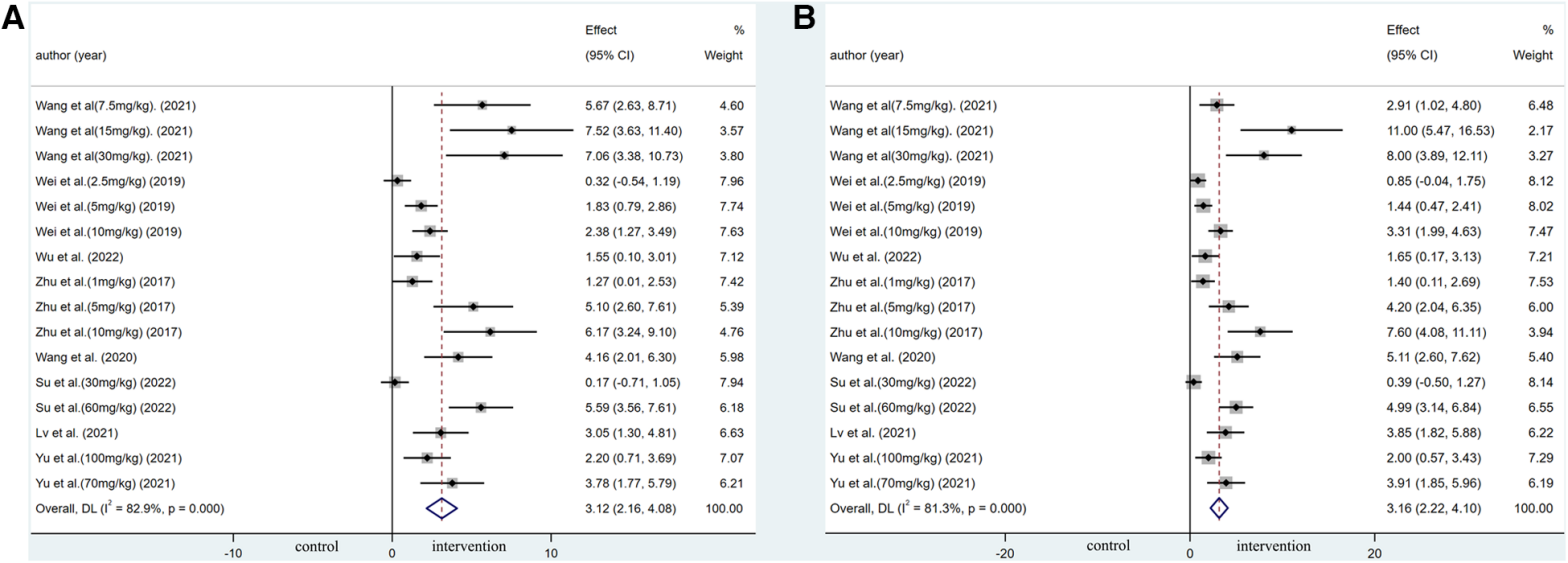
Forest plot of studies investigating the effects of saponins treatment on cardiac function vs. vehicle in myocardial I/R injury. (**A**) Summary WMD of LVEF. (**B**) Summary WMD of LVFS.

**Figure 7:**
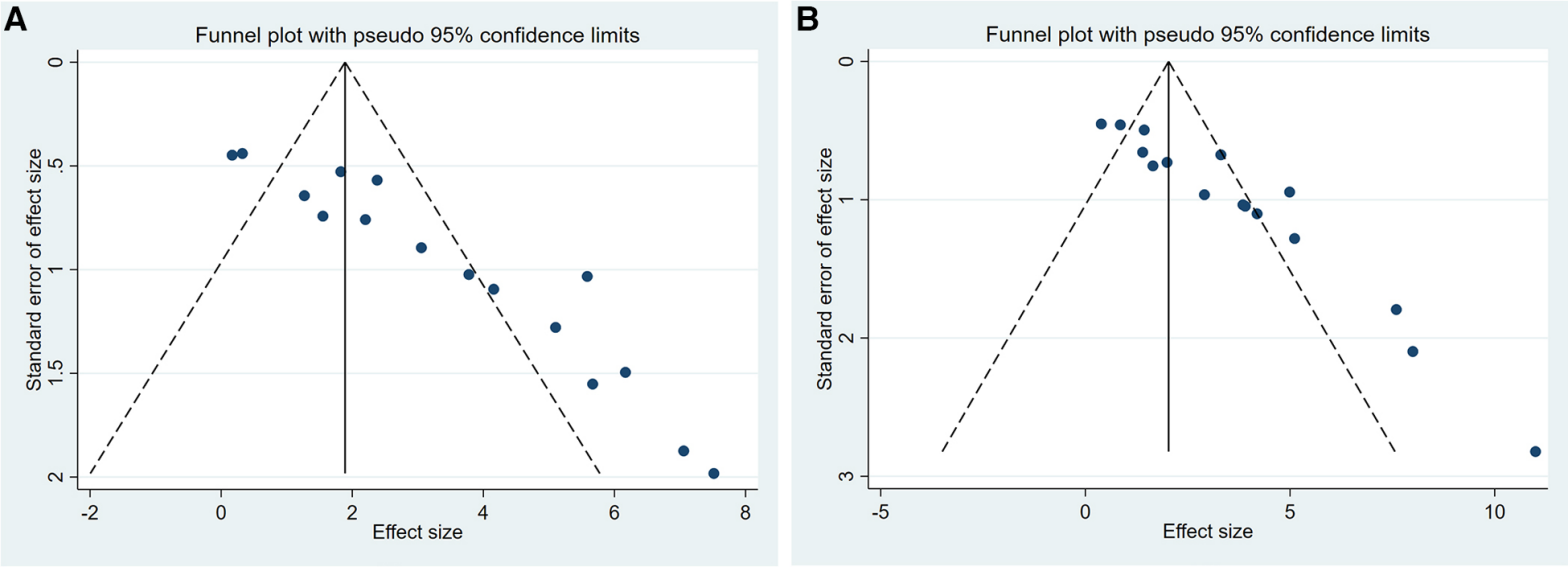
Assessment of publication bias among the studies reporting EF and FS. (**A**) Funnel plot of studies reporting EF. (**B**) Funnel plot of studies reporting FS.

**Figure 8:**
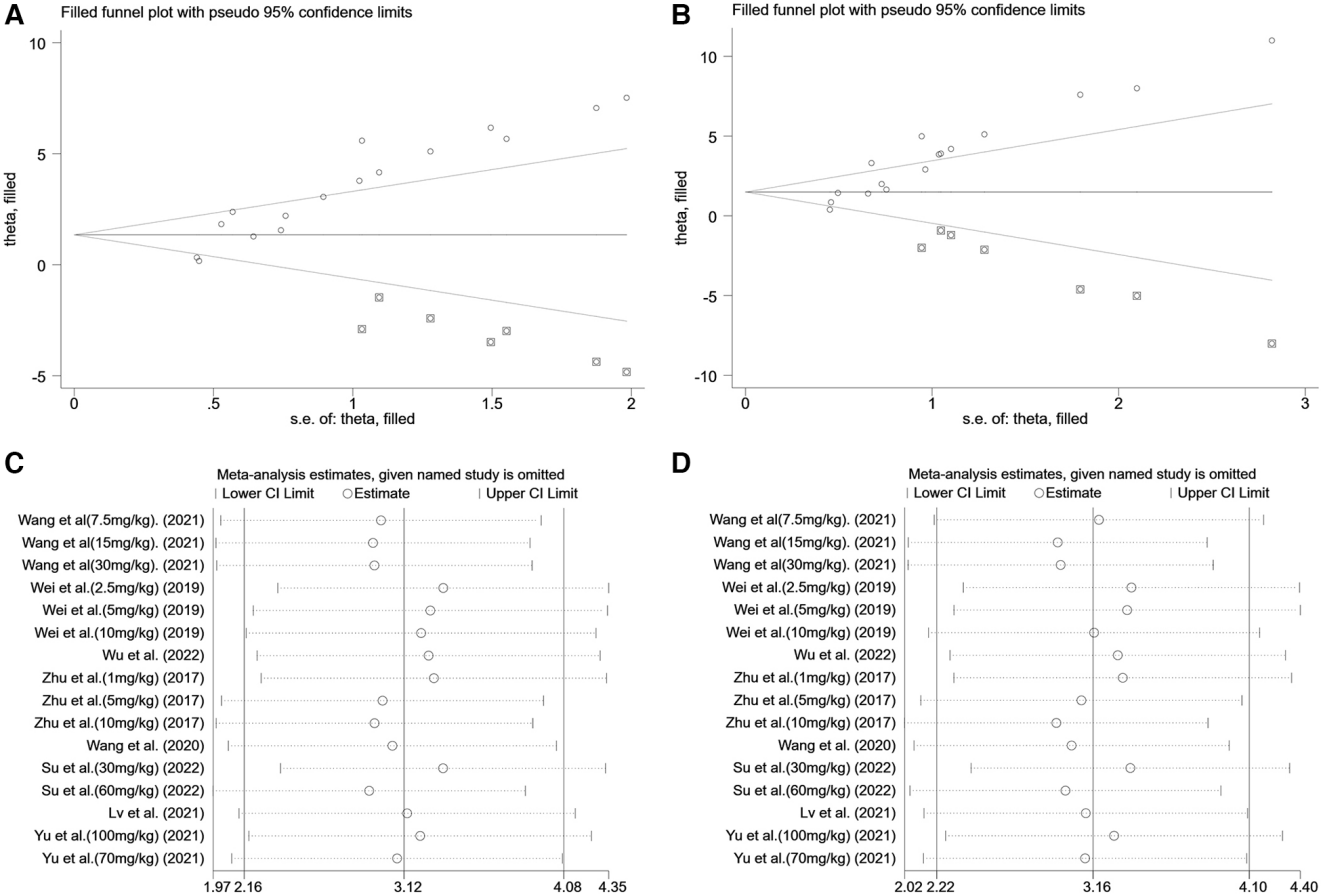
Sensitivity analysis of summary WMD of EF&FS for saponins treatment vs. vehicle in myocardial I/R injury. (**A**) Filled funnel plot with pseudo 95% confidence limits of EF. (**B**) Filled funnel plot with pseudo 95% confidence limits of FS. (**C**) Pooled effect sizes and 95% confidence limits of studies reporting EF after each study were omitted. (**D**) Pooled effect sizes and 95% confidence limits of studies reporting FS after each study were omitted.

**Figure 9:**
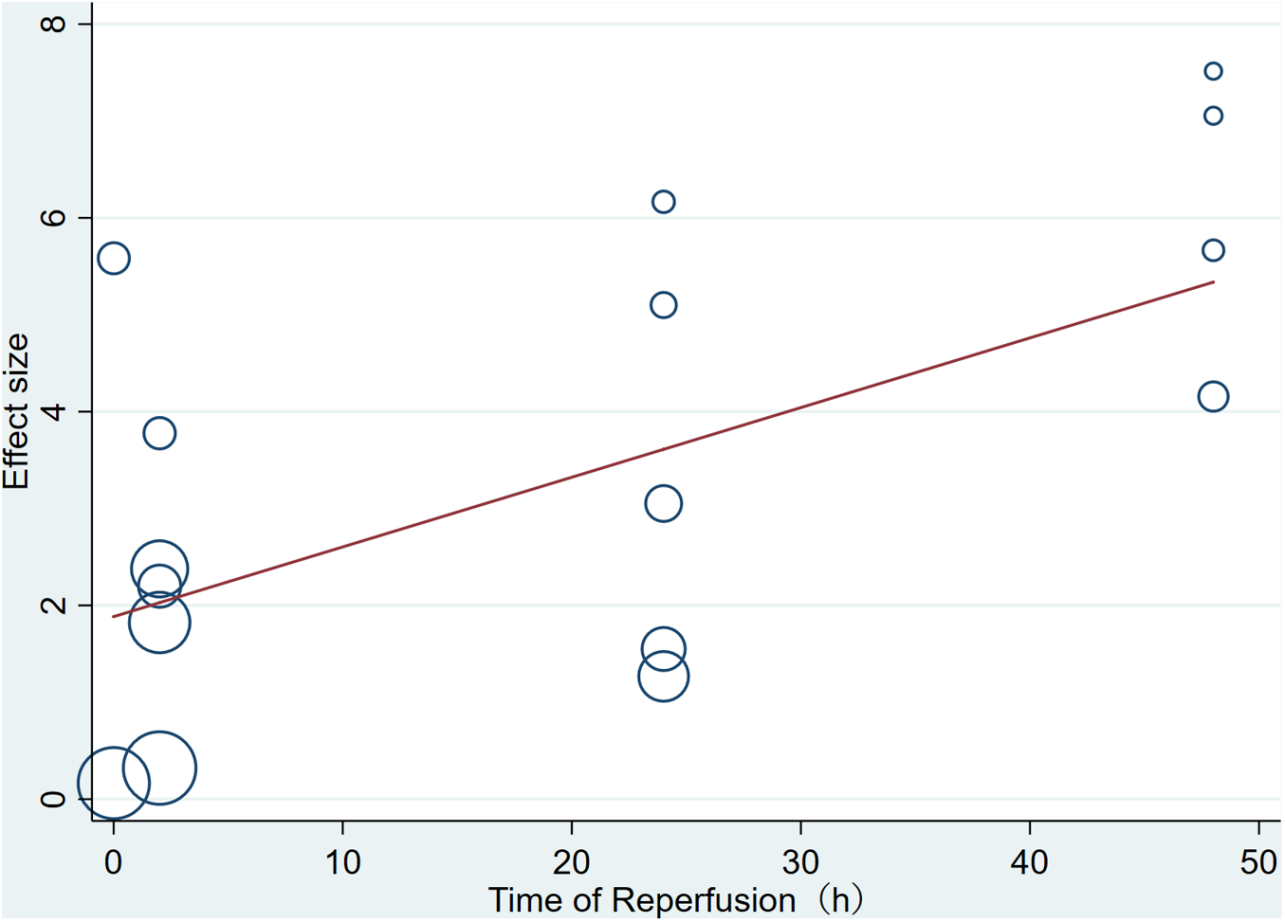
Meta-regression of pooled estimates of EF with the time of reperfusion as the covariate.

Pooled data from 16 arms involving 168 animals showed that saponin treatment significantly increased LVEF after myocardial I/R injury compared with placebo treatment (WMD: 3.119, 95% CI: 2.165 to 4.082, *P *< 0.01) (Figure 6). Heterogeneity was high (*I*^2 ^= 82.9%, *P *< 0.01). The funnel plot and Egger (*P *< 0.001) test showed publication bias (Figure 7). Sensitivity analysis by trim and fill method showed that WMD: 1.548, 95% CI: 0.529 to 2.568, *P *= 0.003. The difference was statistically significant. Systematically deleting each study did not significantly affect WMD and *P* values, indicating that the beneficial effect of saponin treatment on LVEF is stable (Figure 8). Univariate regression showed that the effect size was significantly correlated with reperfusion time (Coefficient: 1.074572, 95% CI: 1.014757 to 1.137913, *P *= 0.017) (Figure 9), suggesting that the inter-study heterogeneity may be mainly due to the differences in the reperfusion time (Table 4).

**Table 4:** The cellular mechanisms of the cardioprotection effect of saponins.

Author	Year	Proposed mechanisms
He et al.	2018	Activating Sirt1 and Nrf2-related signaling pathways and attenuating oxidative stress.
Huang et al.	2021	Attenuating superoxide anion-mediated accumulation of autophagosomes.
Wang et al.	2021	Promoting the interaction between LTCC and BAG3, stabilizing calcium cycling in myocardial cells.
Wang et al.	2018	Alleviating oxidative damage and elevating ATPase activity.
Wei et al.	2019	Increasing phosphorylation of PI3K/AKT and GSK-3β protein and activating PI3K/AKT/GSK-3β signaling pathway.
Wu et al.	2022	Suppressing CTSB/NLRP3- dependent pyroptosis.
Yan et al.	2015	Against apoptosis by activating the AMPK pathway.
Zhang et al.	2017	Inhibit hypoxia/reoxygenation-induced cardiomyocyte apoptosis via Akt activation.
Zhu et al.	2017	Inhibition of mitochondrial oxidative stress and the activation of Nrf2/HO-1mediated anti-apoptosis signaling.
Wang et al.	2020	Alleviated MI/R-induced mitochondrial dysfunction and mitochondrial dynamic imbalance by enhancing OPA1-related mitochondrial fusion via activating AMPK signaling pathways.
Su et al.	2022	Inhibit ERS-induced cell apoptosis, autophagy, oxidative stress, and mitochondrial division.
Lv et al.	2021	Protecting MIRI through SIRT3-dependent regulation of oxidative stress and apoptosis.
Yu et al.	2021	Inhibiting the activation of NLRP3 inflammasome via TLR4/MyD88/NF-*κ*B signaling pathway.
Li et al.	2014	Ameliorating ers-mediated myocardial apoptosis in mice
Wang et al.	2013	Inhibiting excessive ERS-mediated apoptosis
Sun et al.	2014	Anti-oxidation and anti-inflammatory activities.
Yao et al.	2019	Inhibiting the release of inflammatory cytokines and regulating oxidative stress.

#### 3.4.2. Left ventricle fractional shortening percentage

**Figure 10:**
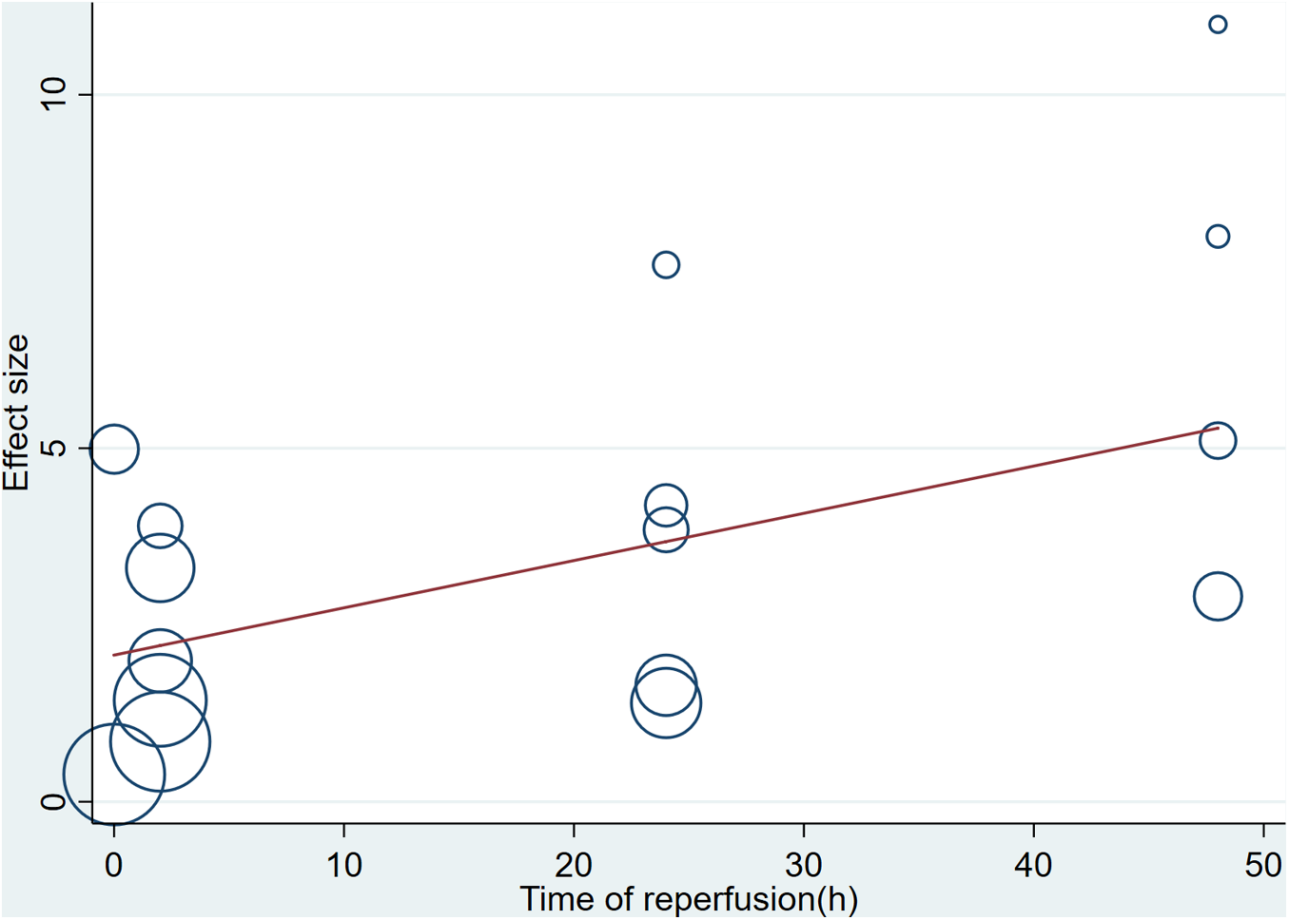
Meta-regression of pooled estimates of FS with the time of reperfusion as the covariate.

A meta-analysis of 16 arms involving 168 experimental animals showed that saponin administration significantly increased LVFS after myocardial I/R injury compared with placebo treatment (WMD: 3.157, 95% CI: 2.218 to 4.097, *P *< 0.001) (Figure 6) with high heterogeneity (*I*^2 ^= 81.3%, *P *< 0.001). The funnel plot and Egger test (*P *< 0.001) showed publication bias (Figure 7). Sensitivity analysis by trim and fill method obtained no significant influence of publication bias on pooled effect size (WMD: 1.697, 95% CI: 0.698 to 2.695, *P *= 0.01). Systematically deleting the WMD and corresponding *P* values did not significantly affect the summary of each study. The results showed that the beneficial effect of saponin administration on FS was stable (Figure 8). Univariate regression showed that the effect size was significantly correlated with the reperfusion time (Coefficient: 1.069146, 95% CI: 1.002232 to 1.140527, *P *= 0.044) (Figure 10), suggesting differences in the reperfusion time may be the primary source of heterogeneity.

### 3.5. Myocardial enzyme

#### 3.5.1. LDH (lactate dehydrogenase)

Pooled data from 20 arms involving 204 animals showed that saponin treatment significantly decreased LDH after myocardial I/R injury compared with placebo treatment (WMD: −4.035, 95% CI: −4.943 to −3.127, *P *< 0.01). Heterogeneity was high (*I*^2 ^= 78.5%, *P *< 0.01). The funnel plot and Egger test (*P *< 0.001) showed publication bias. Sensitivity analysis by trim and fill method showed that WMD: −3.110, 95% CI: −4.029 to −2.190, *P *< 0.001. The result was steady. Systematically deleting each study did not significantly affect WMD and *P* values, indicating that the beneficial effect of saponin treatment on LDH is stable.

#### 3.5.2. CK-MB (creatine kinase MB isoenzyme)

A meta-analysis of 13 arms involving 126 experimental animals showed that saponin administration significantly decreased CK-MB after myocardial I/R injury compared with placebo treatment (WMD: −4.261, 95% CI: −5.520 to −3.002, *P *< 0.001) with high heterogeneity (*I*^2 ^= 79.5%, *P *< 0.001). The funnel plot and Egger test (*P *< 0.001) showed publication bias. Sensitivity analysis by trim and fill method obtained no significant influence of publication bias on pooled effect size (WMD: −3.495, 95% CI: −4.825 to −2.165, *P *< 0.001). Systematically deleting the WMD and corresponding *P* values did not significantly affect the summary of each study. The results showed that the beneficial effect of saponin administration on CK-MB was stable.

## 4. Discussion

So far, this is the first study to systematically collect and quantitatively analyze the protective effects of saponins extract from TCM on myocardial I/R injury. In this study, we systematically reviewed 17 articles involving 26 arms. The results showed that saponins significantly decreased the myocardial infarction size and increased the LVEF and LVFS after myocardial infarction compared with the control group, which proves the positive role of saponins in alleviating MIRI, protecting the myocardium, and promoting the recovery of cardiac function.

PCI and thrombolysis are still the most effective methods for treating ischemia in patients with acute myocardial infarction (31). However, evidence shows that I/R injury after blood flow recovery accounts for nearly half of the infarct area, ultimately aggravating ventricular dysfunction after myocardial infarction (19). Therefore, MIRI is an urgent clinical concern. In recent years, some treatments for myocardial ischemia-reperfusion injury have been partially proven effective, but these methods often have drawbacks that cannot be ignored. For example, one way to reduce reperfusion injury is to inflate/deflate the angioplasty balloon within 1 min after reperfusion. Small-scale trials have shown that infarct size is reduced in patients who receive this treatment. However, large-sample randomized clinical trials for STEMI (ST-segment elevation myocardial infarction) post-processing showed neutral results. And repeatedly inflating the balloon at the lesion site may lead to excessive production of thromboembolism. In recent years, there are also some adjuvant drug interventions for myocardial ischemia-reperfusion, most of which have been proved to be ineffective, but some drugs have shown certain efficacy. For example, cyclosporine-a, metoprolol, glucose modulators, and abciximab, among which cyclosporine and abciximab have well-known side effects, glucose modulators lack prospective clinical trial evidence, and *β*-blockers are also questionable in terms of safety in patients with low systolic blood pressure (<120 mm Hg) and Killip class III patients, limiting their application scenarios. In contrast, as an extract of traditional Chinese medicine, saponins have been widely used by clinicians in China since ancient times. So far, there are no obvious safety problems. This study also confirmed the effectiveness of saponins in reducing ischemia-reperfusion injury in animals. MIRI involves plenty of mechanisms (32). TCM has been used in China for thousands of years. Many of these drugs and therapies have been tested today and have proven superior efficacy (33). Recently, some studies found that saponins from TCM can protect against myocardial ischemia-reperfusion injury through various mechanisms (4, 34–36).

### 4.1. Necrosis

Necrosis is thought to be caused by chemical or physical damage. A decrease in ATP production can cause intracellular calcium overload during myocardial ischemia. During reperfusion, the restoration of energy supply under the premise of calcium overload-induced the oscillatory release and uptake of calcium by the sarcoplasmic reticulum, resulting in uncontrolled excessive muscle fiber contraction and damage to cellular structures. The study of Wang et al. showed that Calenduloside E(CE) could repress L-type calcium current induced by L-type calcium channels(LTCC) agonist and regulate calcium homeostasis (37).

### 4.2. Oxidative stress

After reperfusion, excessive reactive oxygen species (ROS) are produced, which may lead to the oxidation of nucleic acids, lipids, and proteins, further leading to changes in membrane damage, protein function, metabolic disorders, gene mutation, and resulting in oxidative stress (38–41). Studies have shown that Sirt1, Sirt3, and Nrf2 have the function of regulating oxidative stress. He et al. found that Saponins from *Rhizoma Panacis Majoris* (SPRM) protect against MIRI by activating Sirt1 and Nrf2-related signaling pathways and attenuating oxidative stress (10). Tubeimoside I (TBM) reduces the production of ROS by inhibiting NOX2. On the other hand, TBM increased antioxidant factors such as Nrf2 and NQO1 (26). Zhu et al. found that a new flavonoid glycoside (APG) increased SOD activity, decreased H2O2 and MDA, inhibited mitochondrial oxidative stress, and finally attenuated MIRI via activating PKC*ε* signaling (19).

### 4.3. Inflammatory response

After MIRI, neutrophil adhesion and infiltration occur in the coronary artery (5, 42–44). The expression of adhesion molecules increases on the surface of microvascular endothelial cells and leukocytes (9, 45, 46), which promotes adhesion, aggregation, and chemotaxis of neutrophils and resists microcirculation, accelerating the process of MIRI (47). In recent years, the role of NLRP3 inflammatory agents in MIRI has become a research hotspot. NLRP3 can combine with Caspase-1 and Asc to form NLRP3 inflammasome, which requires the activation of NF-*κ*B. Yu et al. found that the pretreatment of *Panax notoginseng* saponins (PQS) significantly down-regulated the expression of NLRP3, ASC, and caspase-1. This effect may be achieved by inhibiting the TLR4/MyD88/NF- *κ* B signal pathway (24).

### 4.4. Intracellular calcium overload

Na^+^-K^+^-ATPase is an essential system of cellular energy metabolism. Na^+^-K^+^-ATPase activity decreased significantly after MIRI (48). This situation increases intracellular Na^+^, and more Ca^2+^ enters the cell through Na^+^-Ca^2+^ exchange, resulting in calcium overload (49–51). Total saponins of *A. Elata* (aralosides, AS) can significantly increase the activity of Na^+^-K^+^-ATPase and Ca^2+^-Mg^2+^-ATPase (17). The study of Wang et al. showed that Calenduloside E(CE) could promote the interaction between LTCC and bcl2-related athanogene 3 (BAG3), reduce MIR-induced calcium overload and play a protective role in the heart (22).

### 4.5. Autophagy

Autophagy is a phenomenon in which cells use lysosomes to digest their organelles or some proteins, and it is the key to maintaining normal metabolism and renewal of some organelles. During myocardial ischemia, autophagy can provide critical nutrients for cell survival and inhibit cell apoptosis and necrosis by digesting non-functional proteins. O^2•−^ is a potential mechanism of autophagy. Huang et al. found that Saponin astragaloside IV(ASIV) could increase SOD2 levels and reduce the accumulation of O^2•−^ and autophagosome, alleviating I/R-induced cell death and protecting cardiomyocytes (13).

### 4.6. Programmed cell death

#### 4.6.1. Apoptosis

Apoptosis is a form of cell death regulated by genes. Once apoptosis is initiated, the volume of apoptotic cells will shrink, the cytoplasm will condense, and finally, apoptotic bodies will be formed. Activation of caspases plays a crucial role in apoptosis. Total saponins extracted from Aralia taibaiensis(sAT) pretreatment significantly inhibited the release of Cyto-c from mitochondria in I/R injured cardiomyocytes, thereby inhibiting the activity and expression of caspase-3 (25). *Apigenin-7-O-β-D-(-6*″*-p-coumaroyl*)-glucopyranoside (APG) is a new flavonoid glycoside isolated from Clematis tangutica. Zhu et al. found that APG preconditioning activated Nrf2/HO-1 signaling, down-regulated Bax and cleaved caspase 3, upregulated Bcl2, and reduced the apoptosis rate of IR-damaged cardiomyocytes (19).

#### 4.6.2. Pyroptosis

Pyroptosis is a new form of programmed cell death with an inflammatory response characterized by forming inflammatory bodies and activating caspase and gasdermin (52). Pyrocytosis mainly depends on NLRP3 or caspase-1 (53). Ilexsaponin I(ISI) is a triterpenoid saponin obtained from *Ilex pubescens Hook. et Arn.* Study by Wu et al. has shown that ISI can promote the formation of the CTSB/HSP70 complex to disturb CTSB/NLRP3 complex, thus inhibiting NLRP3-mediated cell pyrogenesis (23).

### 4.7. Saponins on multiple signalling pathways

In short, the pathogenesis of MIRI begins with calcium overload and oxidative stress caused by sudden changes in the environment (54). Calcium overload and oxidative stress interact with each other (55, 56), resulting in the destruction of cell structure and function (57, 58), leading to necrosis or apoptosis (1). The necrotic cells cause a different inflammatory response, stimulating other cells to start pyroptosis. Saponins can regulate calcium balance by improving membrane ion channel activity, reducing calcium overload, and preventing calcium overload-induced cell death and oxidative stress. At the same time, saponins reduce the production of reactive oxygen species, promote the removal of reactive oxygen species, reduce autophagy (59–61), apoptosis and inflammatory responses caused by oxidative stress, and reduce pyroptosis caused by inflammatory responses (6, 62–64). Therefore, traditional Chinese medicine saponins can act on multiple targets in the process of MIRI and protect MIRI through the interaction of many pathways.

## 5. Limitation

There are some limitations in our meta-analysis. First, the studies we included have differences in selecting experimental materials (65–68). The species, strain, age, body weight, and other characteristics of experimental animals may result in heterogeneity in absorption rate. Second, there is no standard scheme for the duration of ischemia/reperfusion in animal models and the administration regimen of saponins (68–72). Significant differences exist in the dosage and treatment scheme of saponins among the studies, which may result in a high heterogeneity and further affect the interpretation of the results. Through subgroup analysis, we found that sample size may be sources of heterogeneity among studies reporting infarct size. It is worth noting that among the study reporting LVEF and LVFS, we found a linear correlation between reperfusion time and the effect of meta-regression. That is, longer reperfusion time is more beneficial to the recovery of cardiac function after myocardial infarction, and that reperfusion time was the primary source of heterogeneity among studies reporting LVEF and LVFS. Funnel plots and Egger tests showed publication bias in this study. However, after adding potential unpublished studies through the trim and fill method, the results were still stable, indicating that publication bias does not affect the conclusions of this study. The sensitivity analysis data also confirmed the effectiveness and reliability of saponins in improving infarct size after reperfusion injury. The included studies reported the immediate efficacy of saponins in improving infarct size and inhibiting secondary cardiac dysfunction. However, one of the disadvantages of traditional Chinese medicine and its extracts is that their components are complex, and their metabolic kinetics in various species is not fully understood. Whether saponins extracted from TCM can maintain their cardioprotective effect for longer is unclear and needs further exploration. Finally, more extensive animal studies have not confirmed the beneficial effects of saponins extracted from TCM. Therefore, there is an urgent need for further research on large animals before human clinical trials.

## 6. Conclusion and perspectives

This systematic review demonstrated that saponins extracted from TCM protect MIRI by significantly reducing infarct size and improving cardiac function. It provides a theoretical basis for the clinical application of saponins extracted from TCM combined with immediate coronary artery revascularization in treating acute myocardial infarction. The study of saponins against MIRI is still in the initial stage. A systematic summary of the anti-MIRI mechanism of saponins could lay the foundation for studying anti-MIRI effects and structure-activity relationships of saponins, thus contributing to developing anti-MIRI drugs with new mechanisms or new targets. Therefore, studying saponins will play an essential role in developing anti-MIRI drugs. Further research on the intervention of traditional Chinese medicine saponins should consider the use of large animals and more standardized and strict experimental procedures to draw more reliable conclusions to promote its application.

## Data Availability

The original contributions presented in the study are included in the article/Supplementary Material, further inquiries can be directed to the corresponding author.
