# Supplementary figures and images for: Effect and possible mechanisms of saponins in Chinese herbal medicine exerts for the treatment of myocardial ischemia-reperfusion injury in experimental animal: a systematic review and meta-analysis
Source: Front Cardiovasc Med. 2023 Jul 26;10:1147740. doi: 10.3389/fcvm.2023.1147740 (PMC10410164; doi:10.3389/fcvm.2023.1147740)

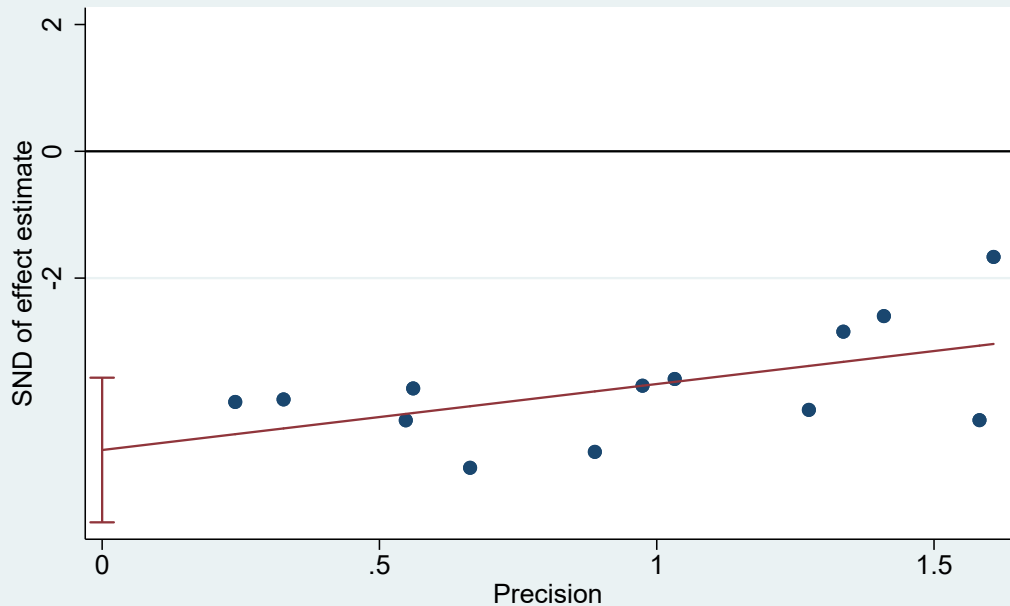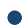

Study

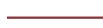

regression line

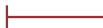

95% CI for intercept

Supplement: Supplementary file 1 [file Presentation1.zip › Presentation1/Supplementary Figures/CKMB Eggers.pdf]

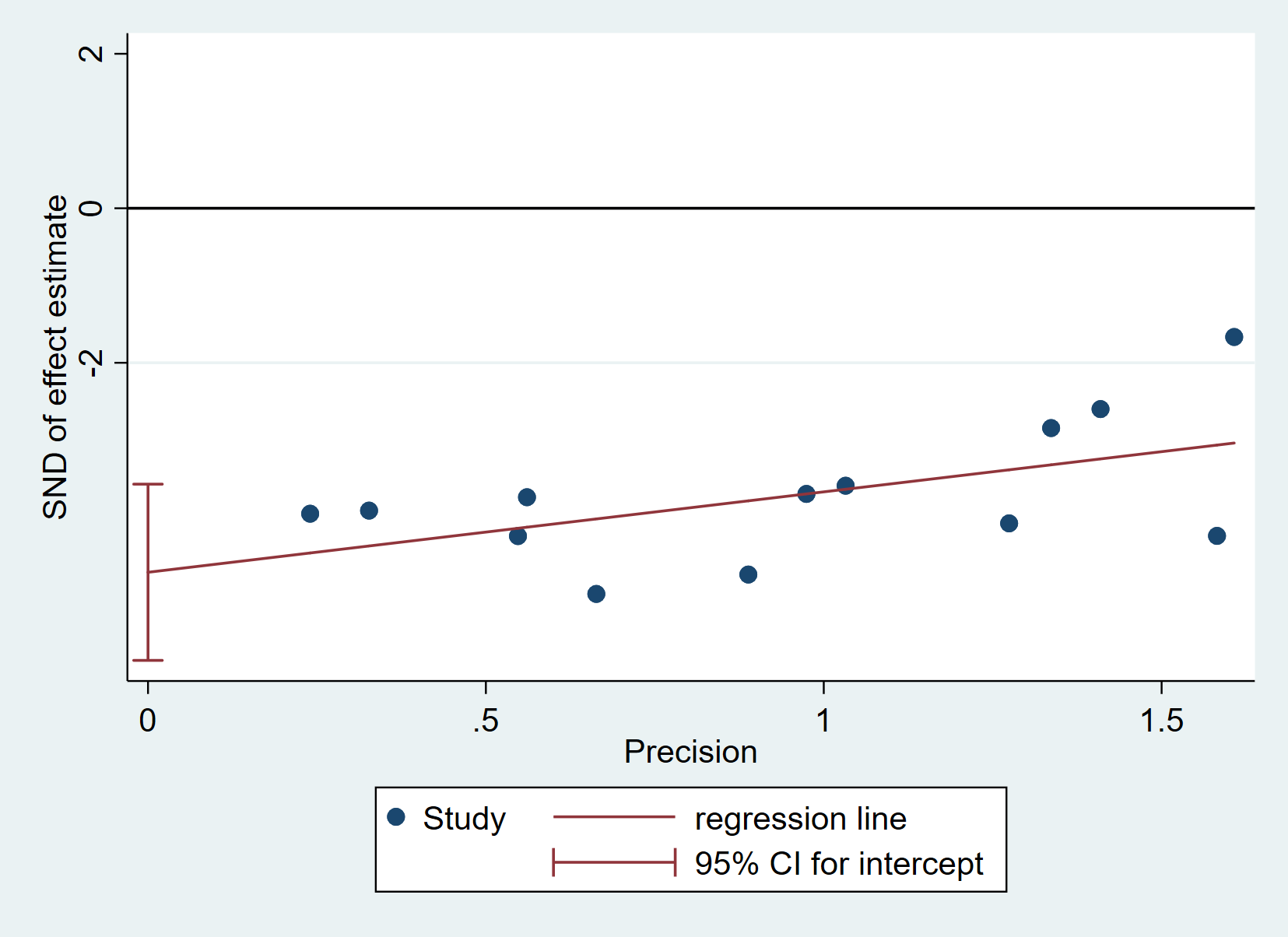

Supplement: Supplementary file 1 [file Presentation1.zip › Presentation1/Supplementary Figures/CKMB Eggers.tif]

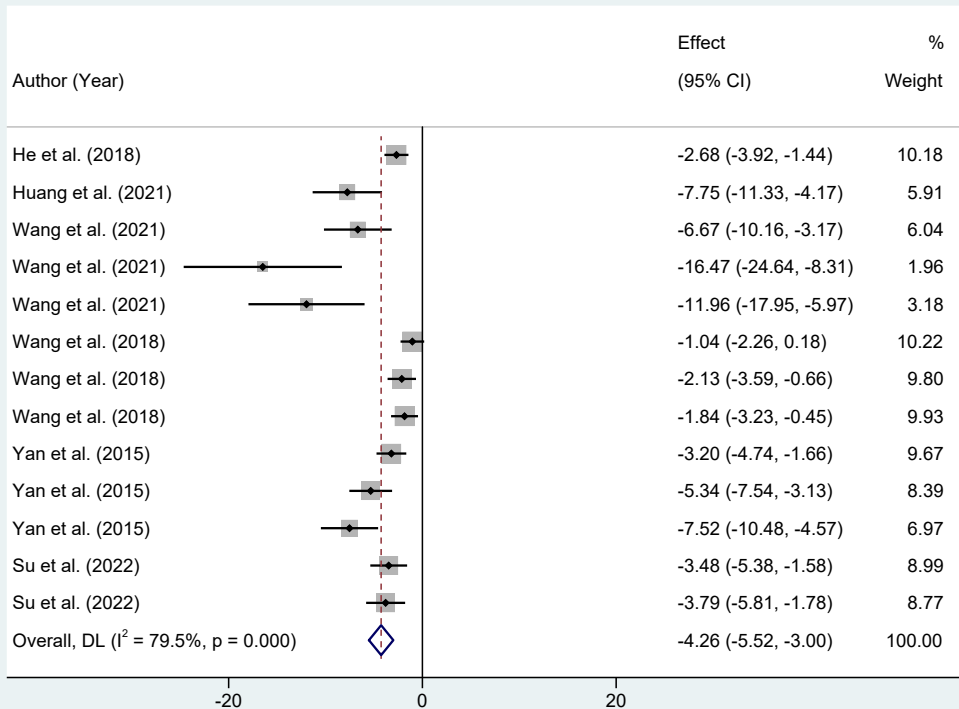

Supplement: Supplementary file 1 [file Presentation1.zip › Presentation1/Supplementary Figures/CKMB forest.pdf]

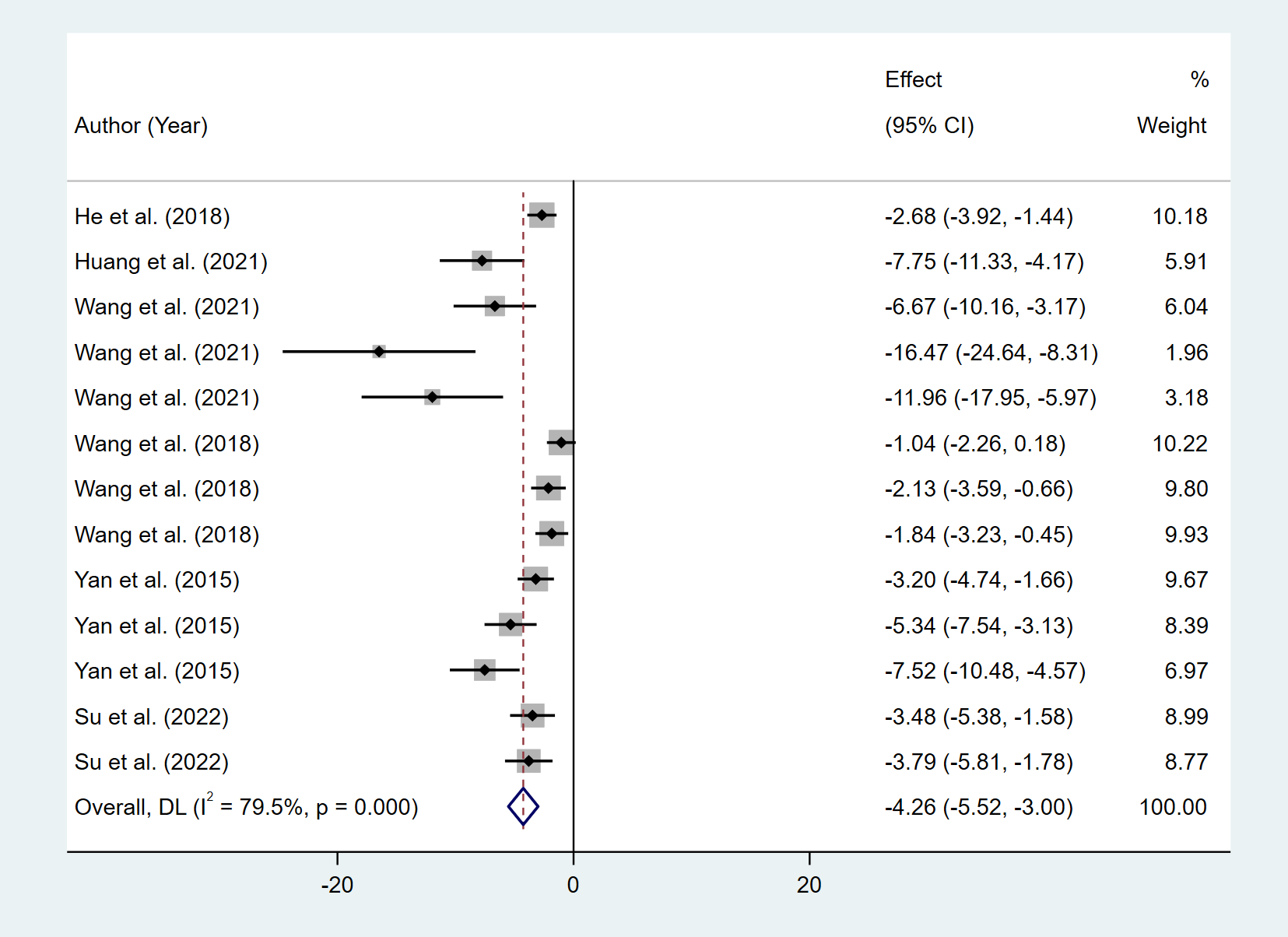

Supplement: Supplementary file 1 [file Presentation1.zip › Presentation1/Supplementary Figures/CKMB forest.tif]

Funnel plot with pseudo 95% confidence limits

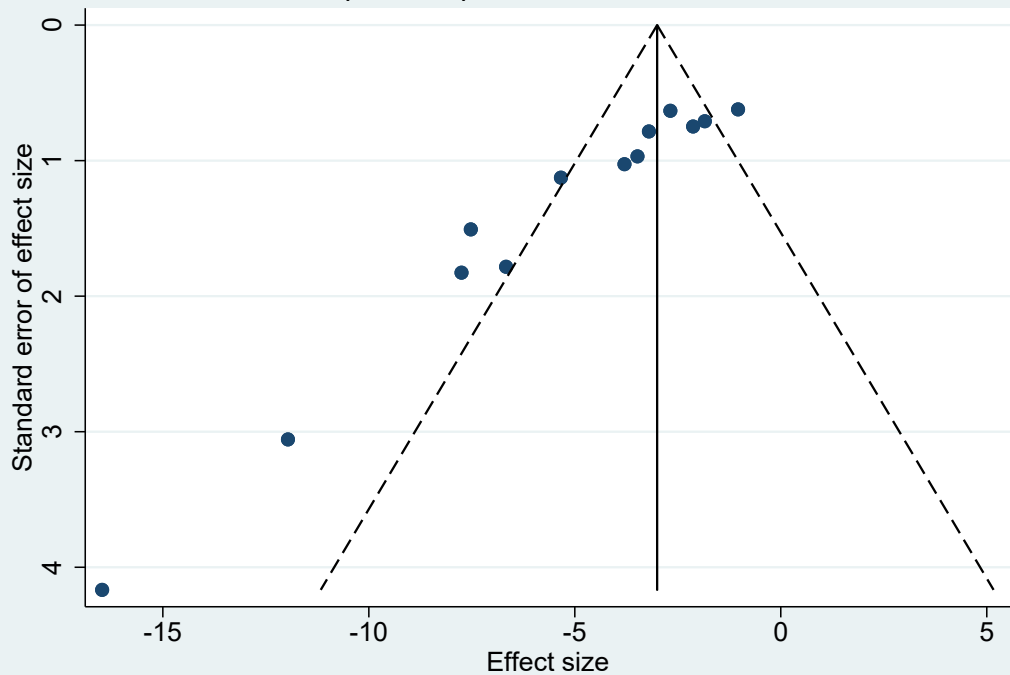

Supplement: Supplementary file 1 [file Presentation1.zip › Presentation1/Supplementary Figures/CKMB Funnel.pdf]

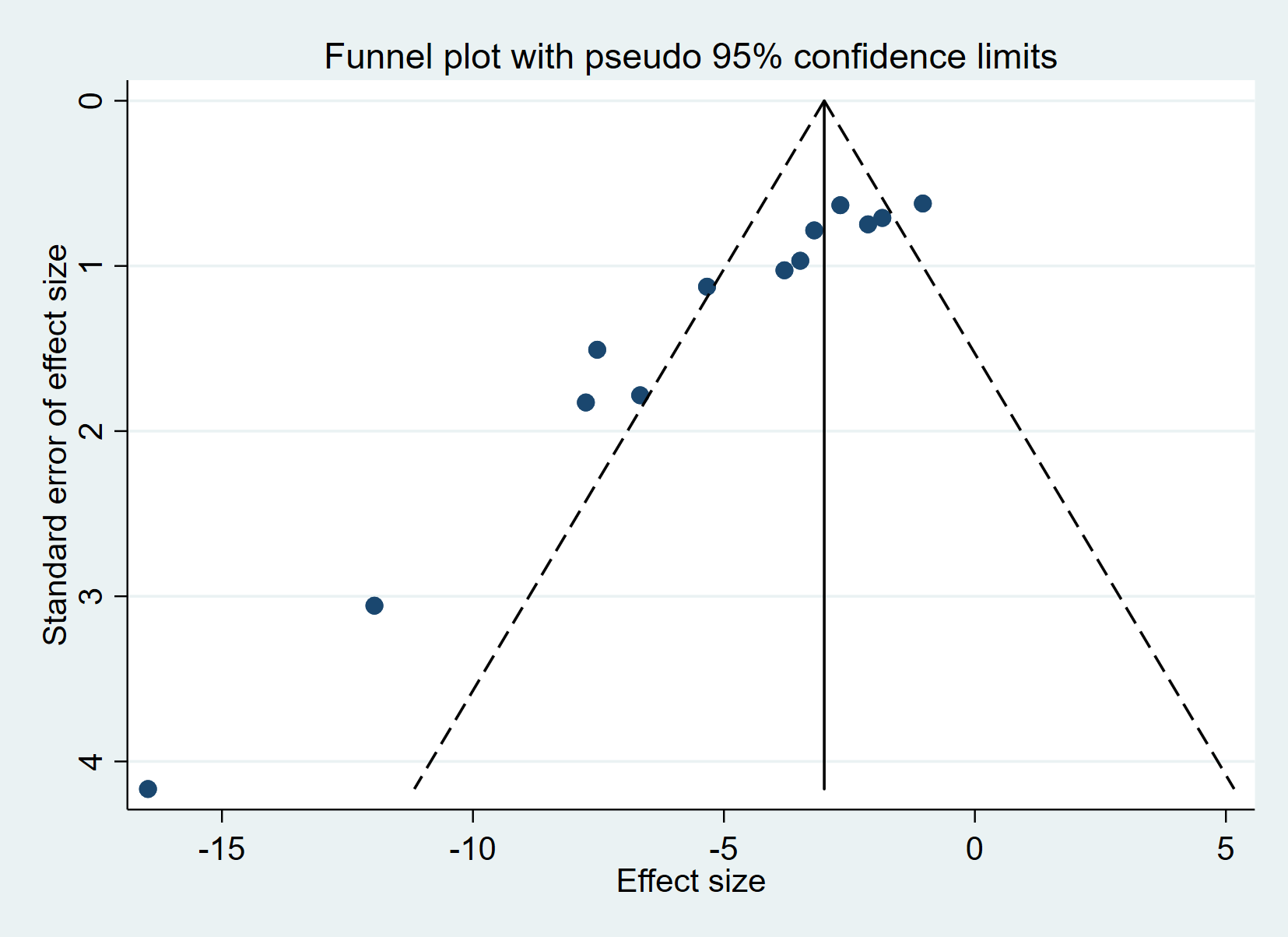

Supplement: Supplementary file 1 [file Presentation1.zip › Presentation1/Supplementary Figures/CKMB Funnel.tif]

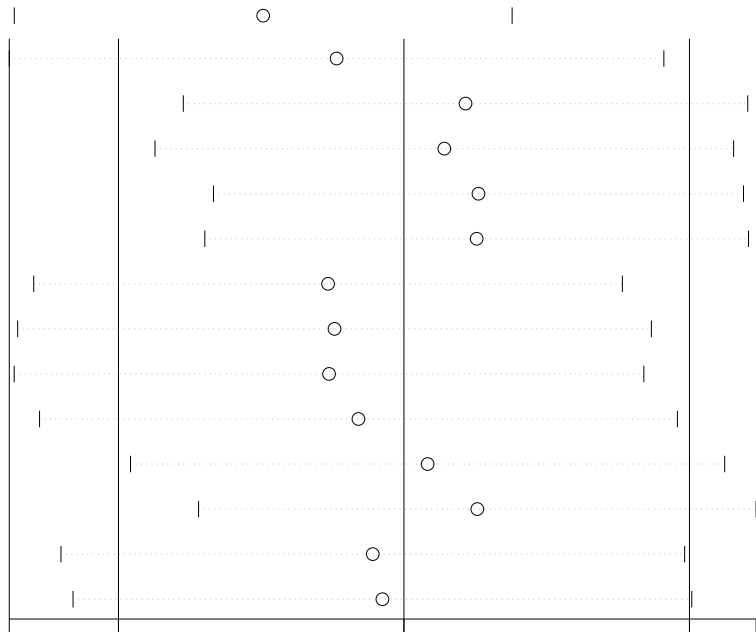

Supplement: Supplementary file 1 [file Presentation1.zip › Presentation1/Supplementary Figures/CKMB inf.pdf]

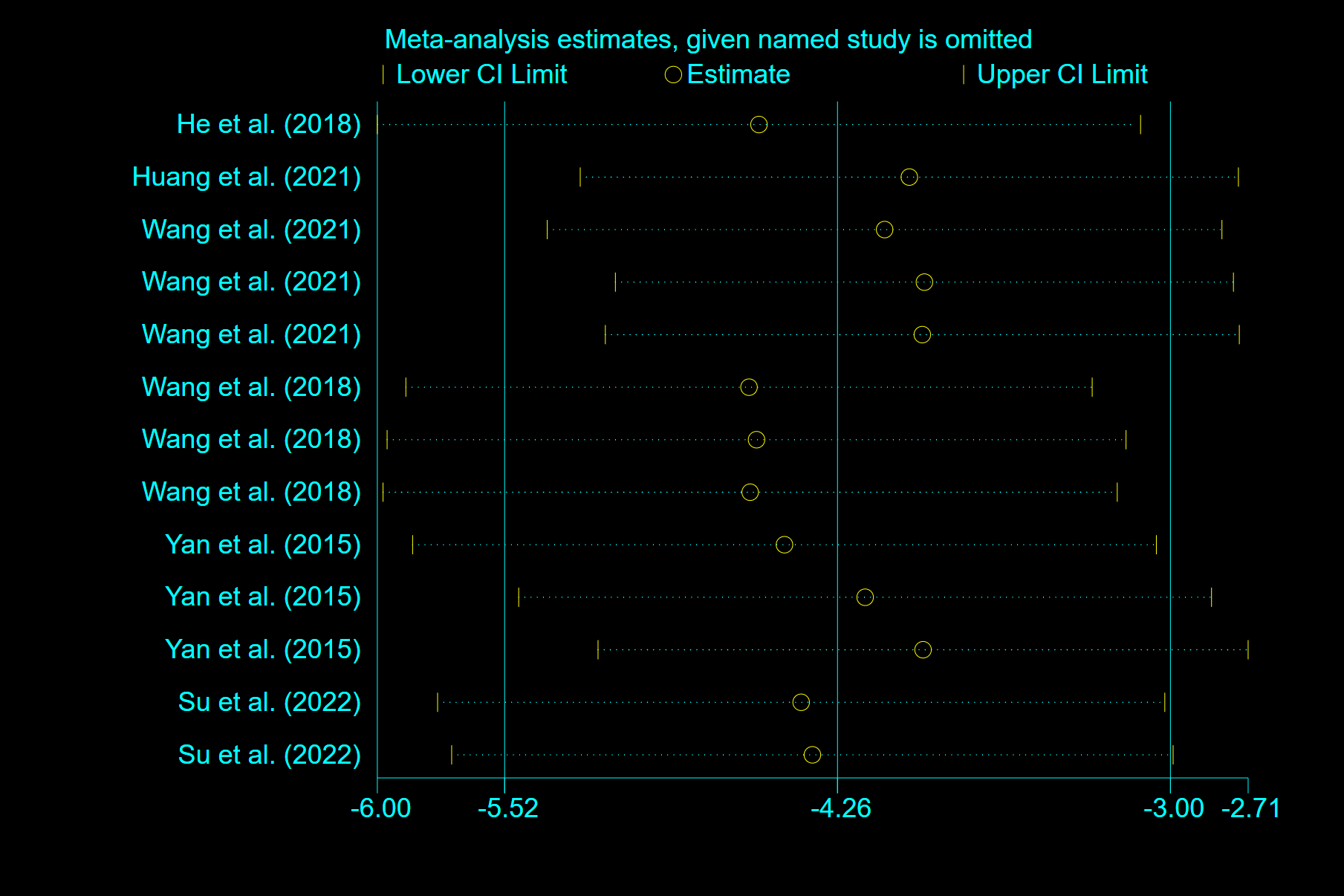

Supplement: Supplementary file 1 [file Presentation1.zip › Presentation1/Supplementary Figures/CKMB inf.tif]

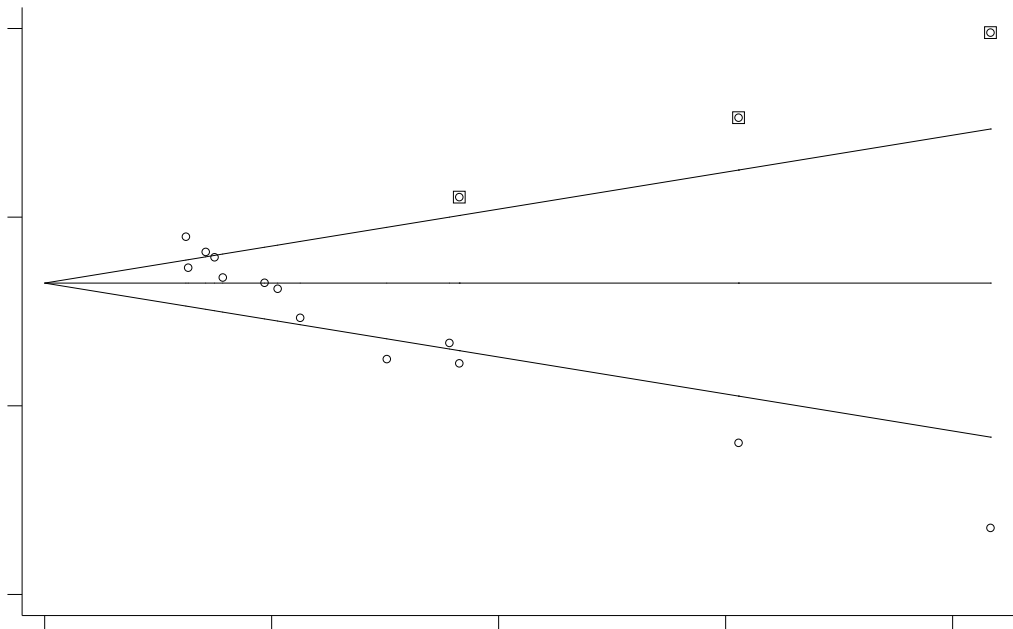

Supplement: Supplementary file 1 [file Presentation1.zip › Presentation1/Supplementary Figures/CKMB Trim&Fill.pdf]

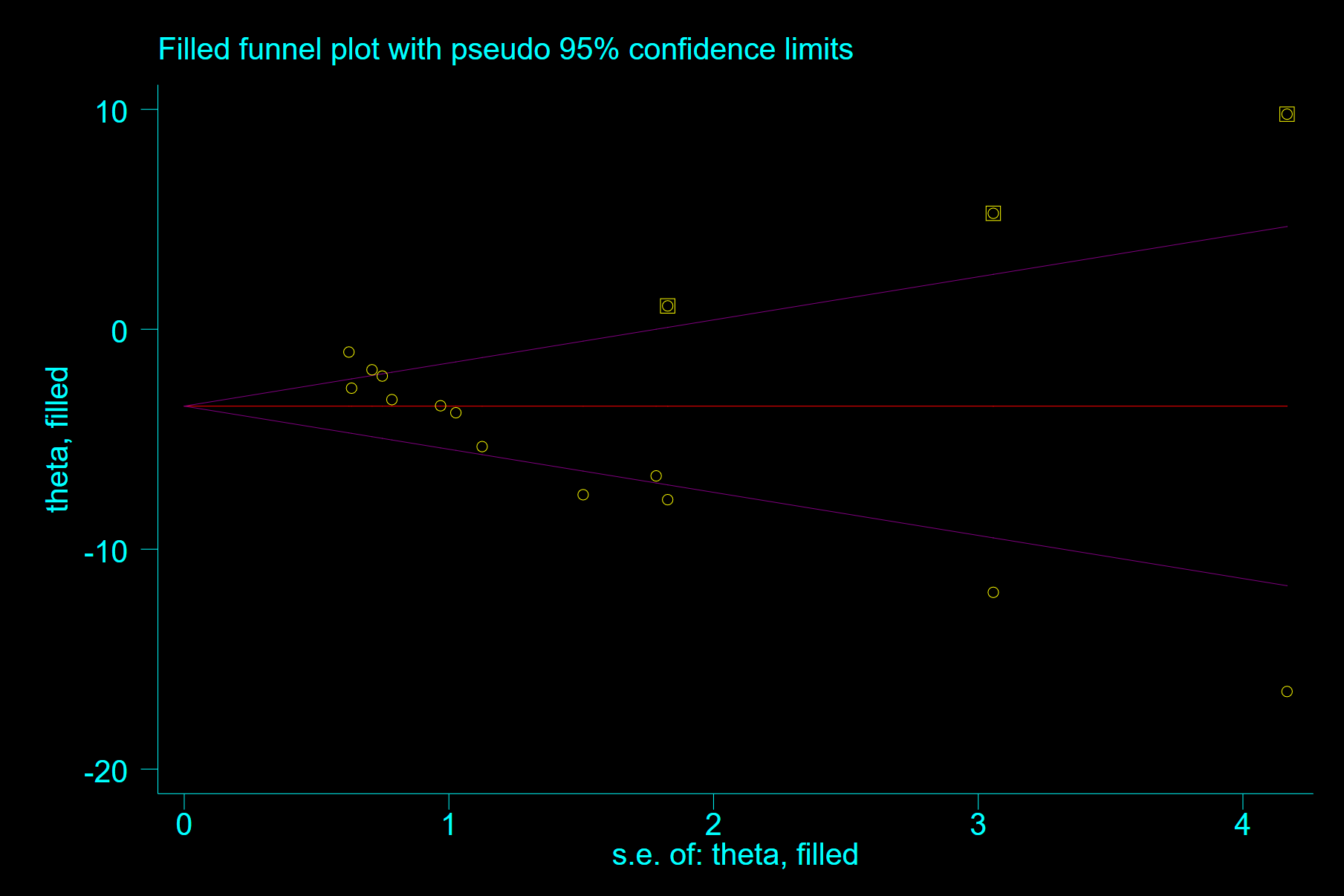

Supplement: Supplementary file 1 [file Presentation1.zip › Presentation1/Supplementary Figures/CKMB Trim&Fill.tif]

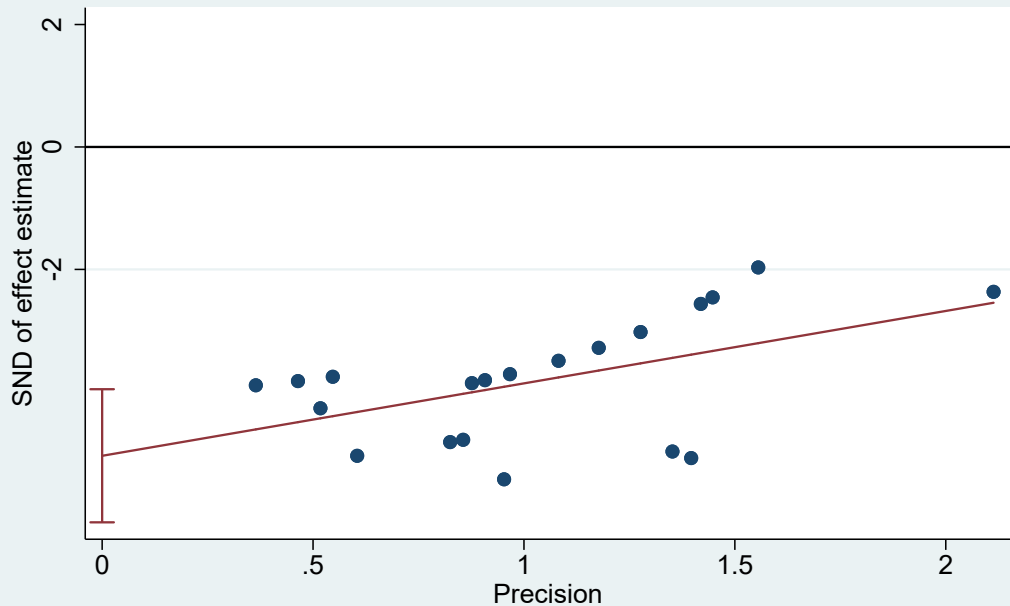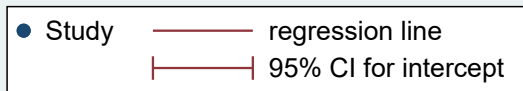

Supplement: Supplementary file 1 [file Presentation1.zip › Presentation1/Supplementary Figures/LDH Eggers.pdf]

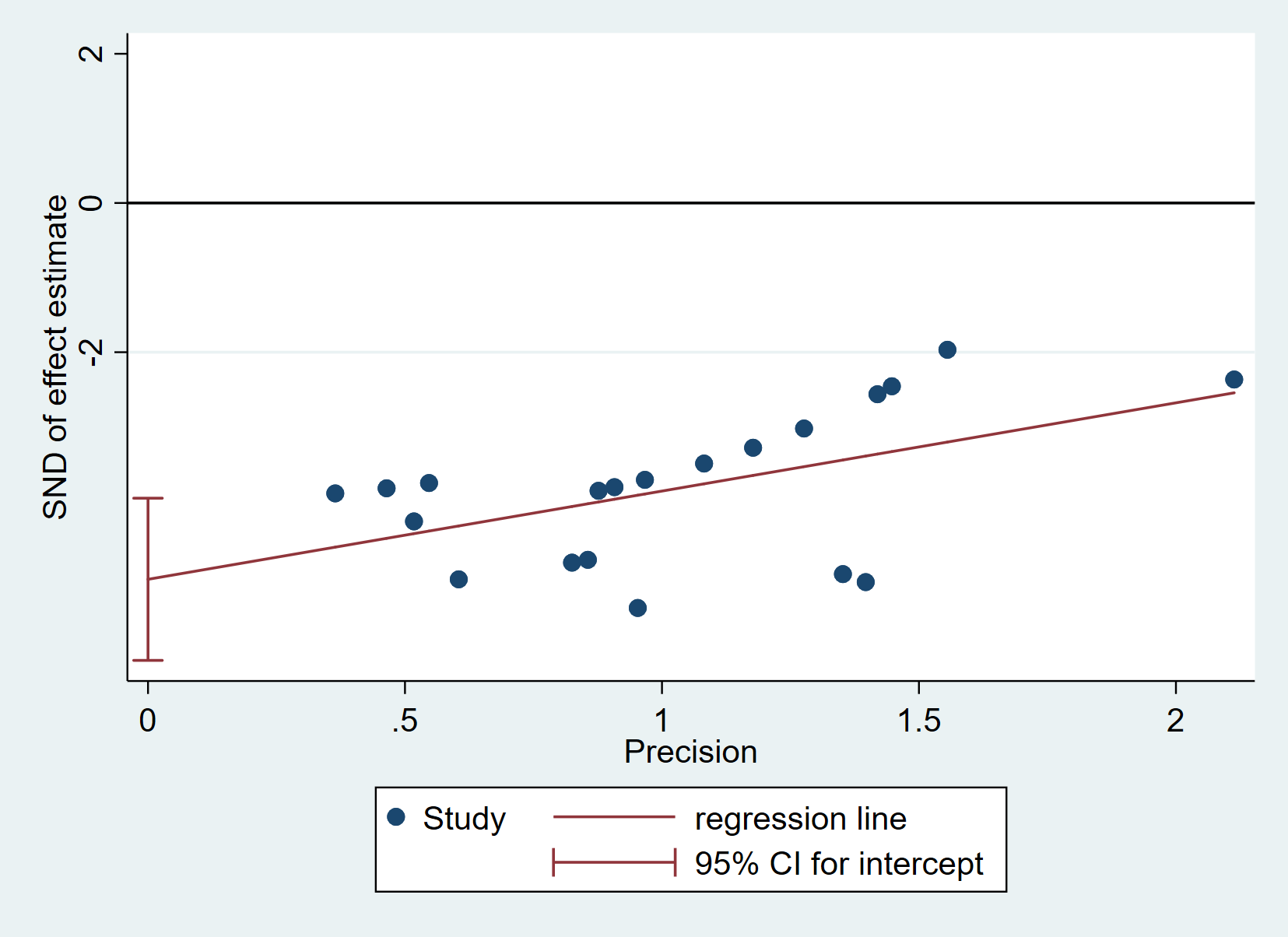

Supplement: Supplementary file 1 [file Presentation1.zip › Presentation1/Supplementary Figures/LDH Eggers.tif]

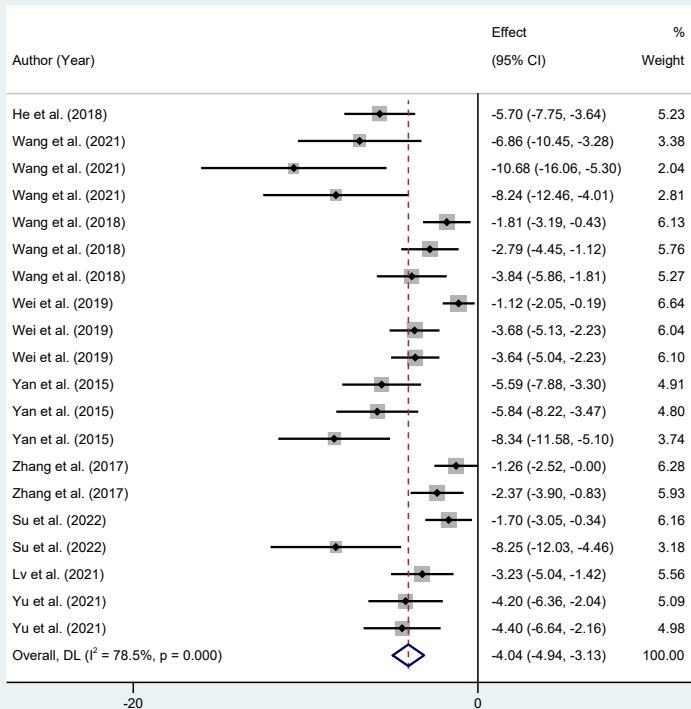

Supplement: Supplementary file 1 [file Presentation1.zip › Presentation1/Supplementary Figures/LDH forest.pdf]

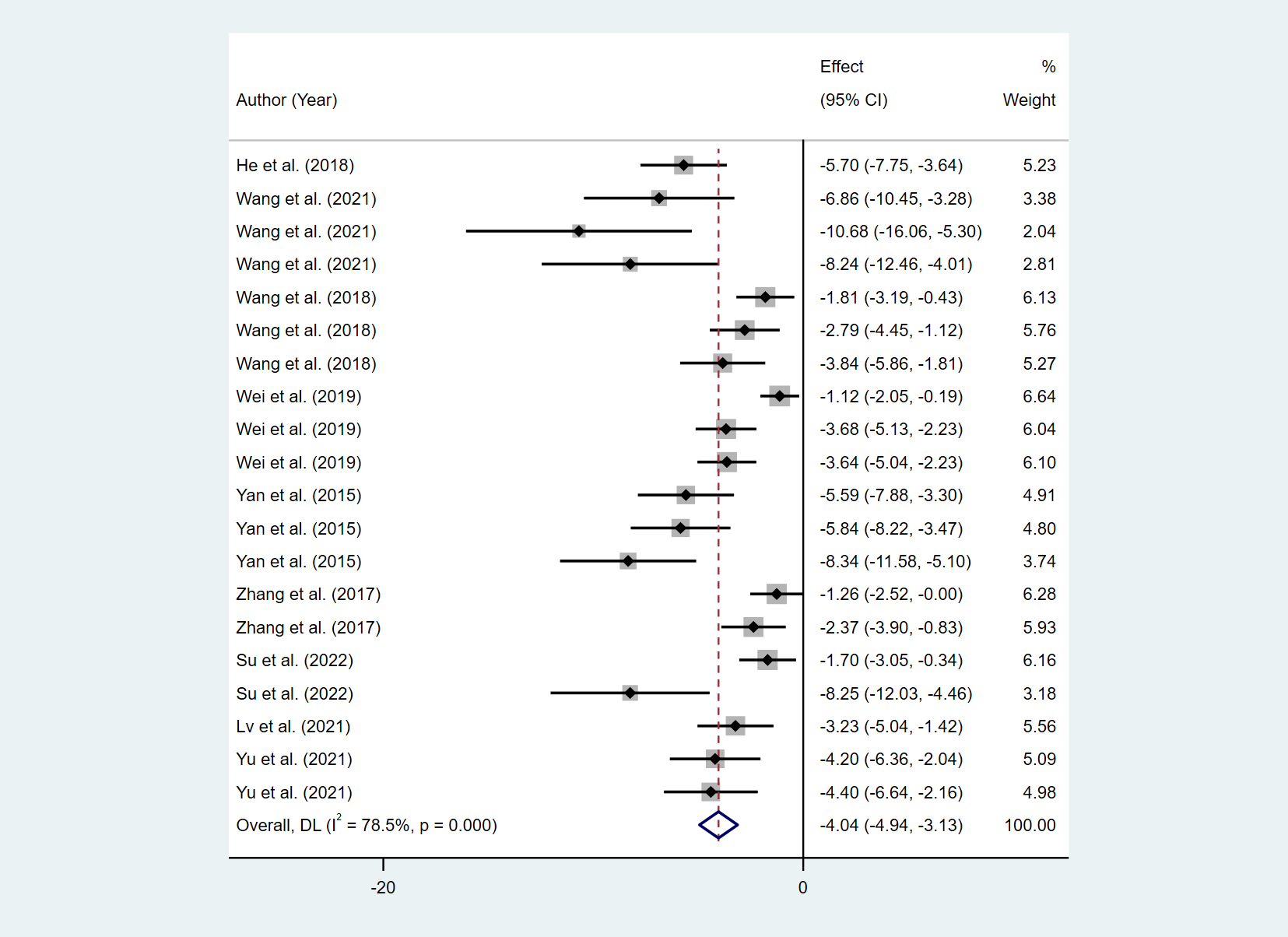

Supplement: Supplementary file 1 [file Presentation1.zip › Presentation1/Supplementary Figures/LDH forest.tif]

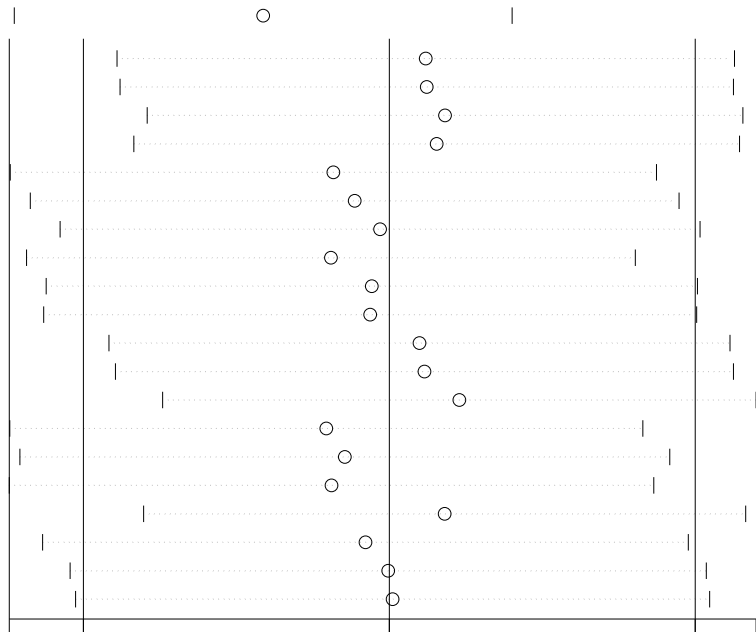

Supplement: Supplementary file 1 [file Presentation1.zip › Presentation1/Supplementary Figures/LDH inf.pdf]

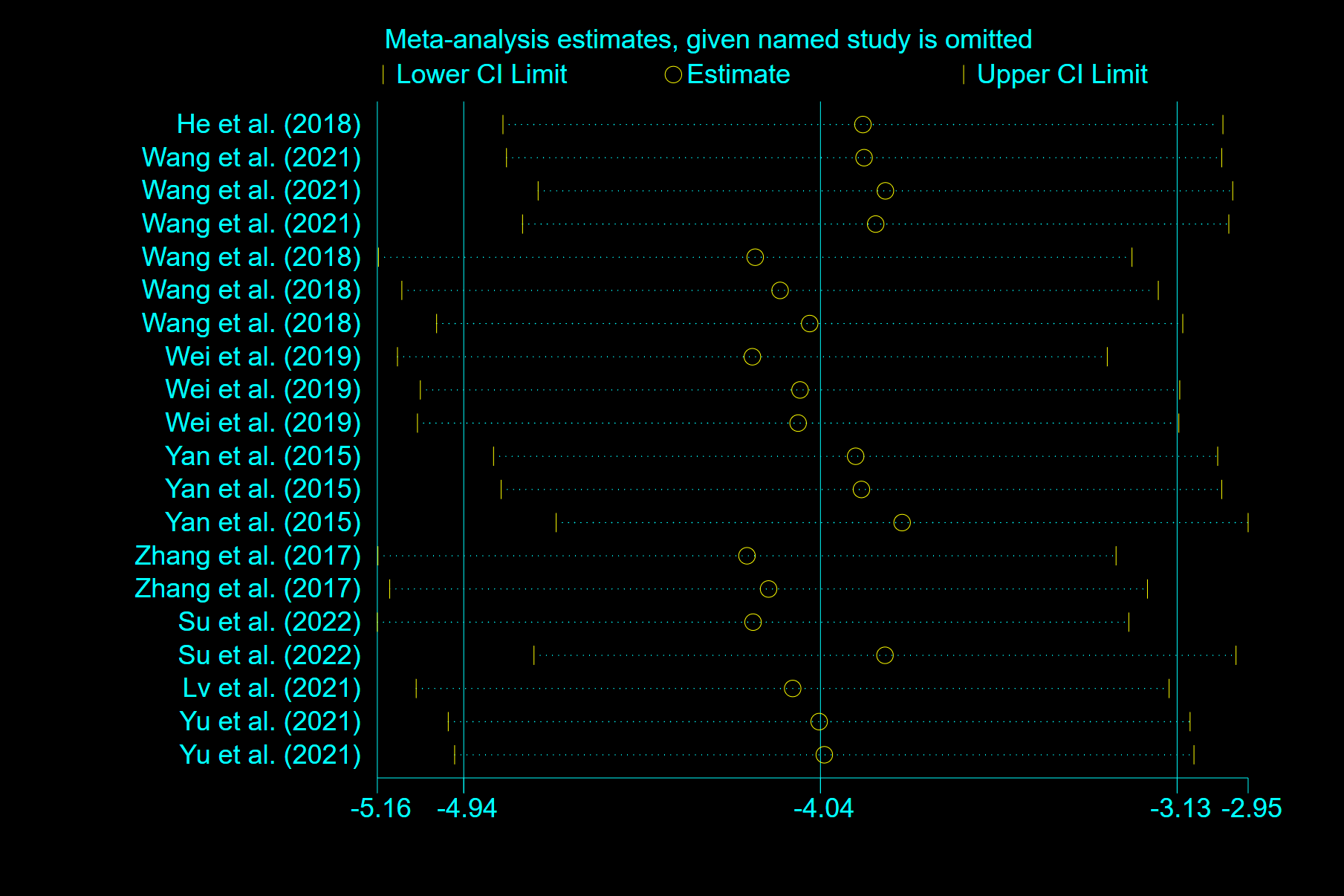

Supplement: Supplementary file 1 [file Presentation1.zip › Presentation1/Supplementary Figures/LDH inf.tif]

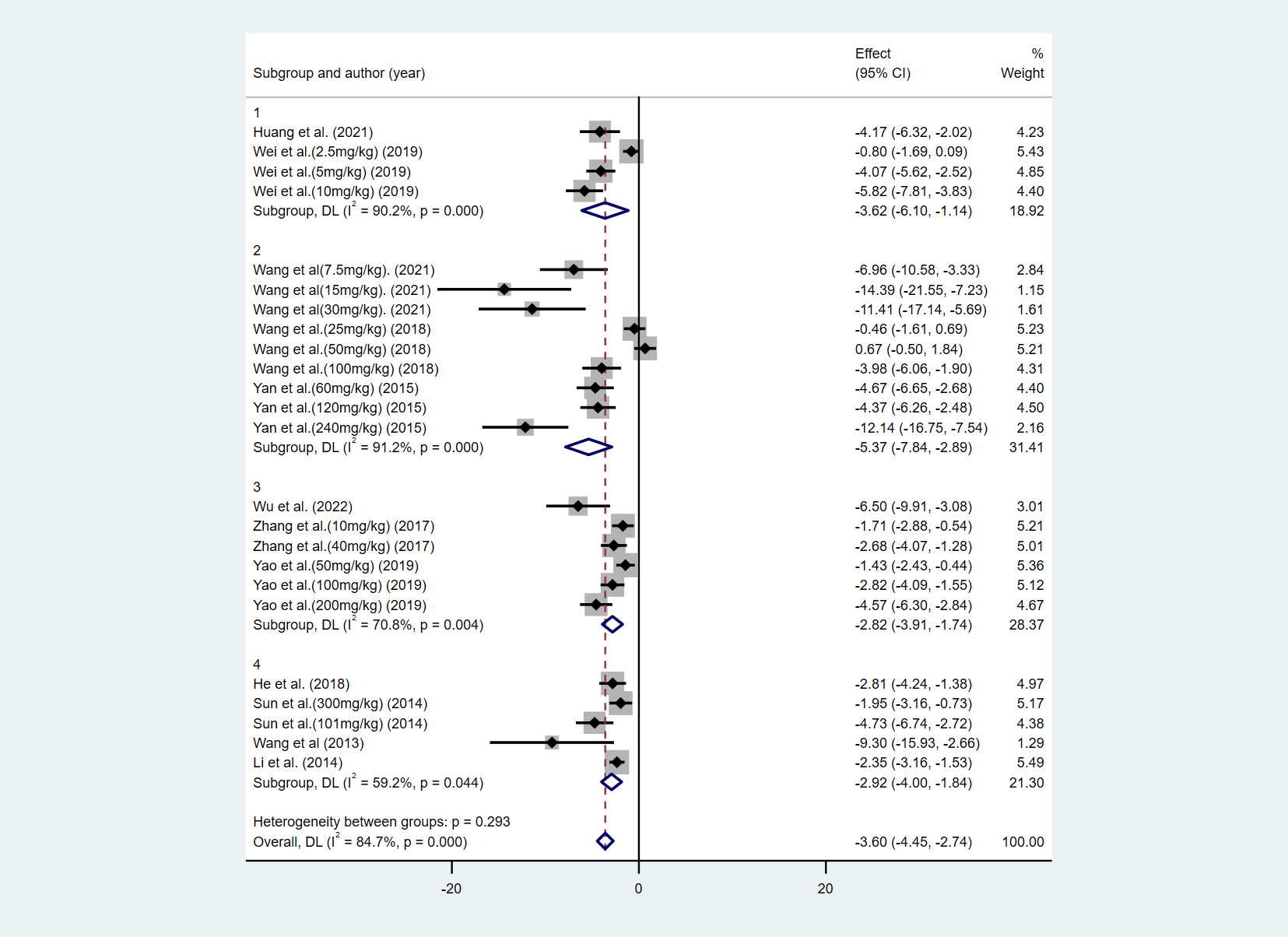

Supplement: Supplementary file 1 [file Presentation1.zip › Presentation1/Supplementary Figures/sub group-plant source.tif]

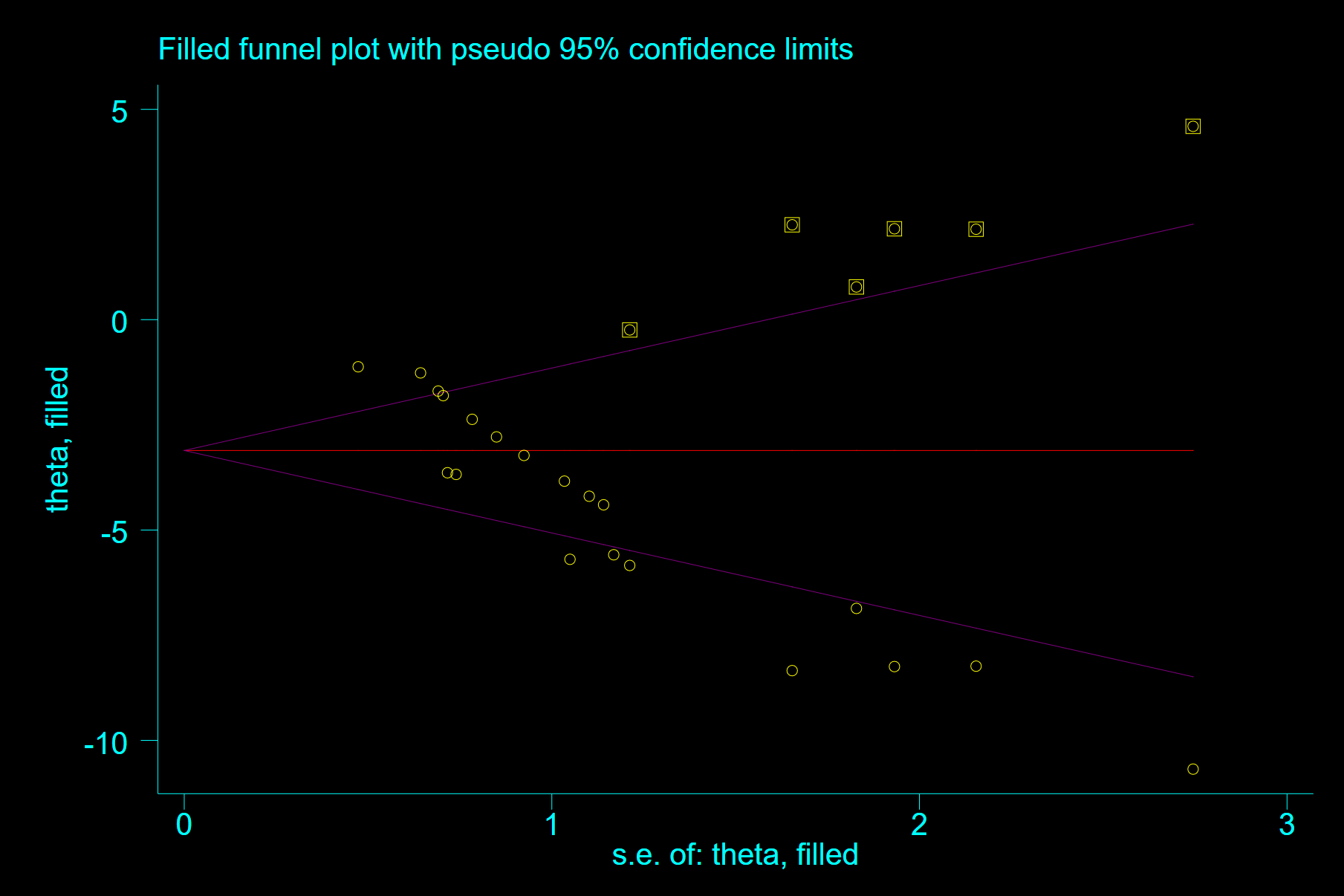

Supplement: Supplementary file 1 [file Presentation1.zip › Presentation1/Supplementary Figures/Trim & Fill.tif]

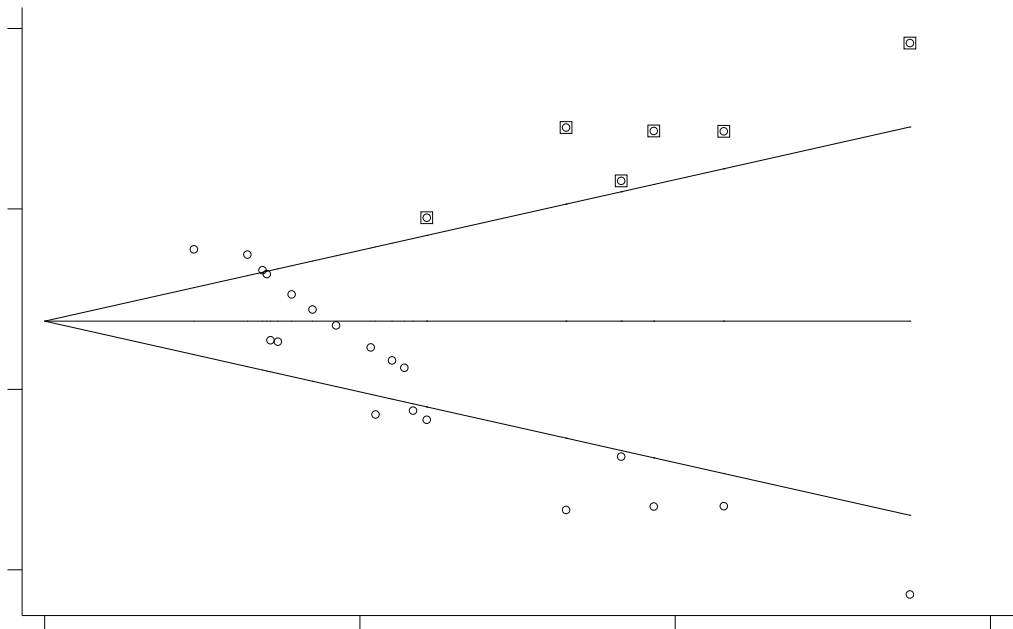

Supplement: Supplementary file 1 [file Presentation1.zip › Presentation1/Supplementary Figures/Trim&Fill.pdf]
